# MSUT2 regulates tau spreading via adenosinergic signaling mediated ASAP1 pathway in neurons

**DOI:** 10.1007/s00401-024-02703-3

**Published:** 2024-03-12

**Authors:** Hong Xu, Qi Qiu, Peng Hu, Kevt’her Hoxha, Elliot Jang, Mia O’Reilly, Christopher Kim, Zhuohao He, Nicholas Marotta, Lakshmi Changolkar, Bin Zhang, Hao Wu, Gerard D. Schellenberg, Brian Kraemer, Kelvin C. Luk, Edward B. Lee, John Q. Trojanowski, Kurt R. Brunden, Virginia M.-Y. Lee

**Affiliations:** 1 Department of Pathology and Laboratory Medicine, Institute on Aging and Center for Neurodegenerative Disease Research, Perelman School of Medicine, University of Pennsylvania, Philadelphia, PA USA; 2 Department of Genetics, Penn Epigenetics Institute, Institute of Regenerative Medicine, University of Pennsylvania, Philadelphia, PA USA; 3 Key Laboratory of Exploration and Utilization of Aquatic Genetic Resources (Ministry of Education), Shanghai Ocean University, Shanghai, China; 4 Interdisciplinary Research Center On Biology and Chemistry, Shanghai Institute of Organic Chemistry, Chinese Academy of Sciences, Shanghai, 201210 China; 5 University of the Chinese Academy of Sciences, Beijing, 100049 China; 6 Department of Pathology and Laboratory Medicine, Penn Neurodegeneration Genomics Center, Perelman School of Medicine, University of Pennsylvania, Philadelphia, PA USA; 7 Geriatric Research Education and Clinical Center, Veterans Affairs Puget Sound Health Care System, Seattle, WA 98108 USA; 8 Department of Psychiatry and Behavioral Sciences, University of Washington School of Medicine, Seattle, WA 98195 USA; 9 Division of Gerontology and Geriatric Medicine, Department of Medicine, University of Washington School of Medicine, Seattle, WA 98104 USA; 10 Translational Neuropathology Research Laboratory, Department of Pathology and Laboratory Medicine, Perelman School of Medicine, University of Pennsylvania, Philadelphia, PA USA

**Keywords:** Tau, Tau spreading, MSUT2, A1AR, ASAP1

## Abstract

Inclusions comprised of microtubule-associated protein tau (tau) are implicated in a group of neurodegenerative diseases, collectively known as tauopathies, that include Alzheimer’s disease (AD). The spreading of misfolded tau “seeds” along neuronal networks is thought to play a crucial role in the progression of tau pathology. Consequently, restricting the release or uptake of tau seeds may inhibit the spread of tau pathology and potentially halt the advancement of the disease. Previous studies have demonstrated that the Mammalian Suppressor of Tauopathy 2 (MSUT2), an RNA binding protein, modulates tau pathogenesis in a transgenic mouse model. In this study, we investigated the impact of MSUT2 on tau pathogenesis using tau seeding models. Our findings indicate that the loss of MSUT2 mitigates human tau seed-induced pathology in neuron cultures and mouse models. In addition, MSUT2 regulates many gene transcripts, including the Adenosine Receptor 1 (A1AR), and we show that down regulation or inhibition of A1AR modulates the activity of the “ArfGAP with SH3 Domain, Ankyrin Repeat, and PH Domain 1 protein” (ASAP1), thereby influencing the internalization of pathogenic tau seeds into neurons resulting in reduction of tau pathology.

**Supplementary Information:**

The online version contains supplementary material available at 10.1007/s00401-024-02703-3.

## Introduction

Tau is a microtubule-associated protein that promotes the polymerization and stabilization of microtubules and modulates the binding of motor proteins involved in axonal transport [81]. The accumulation of misfolded tau deposits is a pathologic hallmark of tauopathy, a group of neurodegenerative disorders of which Alzheimer’s disease (AD) is the most common, affecting more than 10% of the population older than 60 [20, 38]. The abundance of tau pathology in human patients closely correlates with clinical manifestations, including cognitive status and the extent of brain atrophy [3, 71, 76]. Evidence suggests that tau loss- and gain-of-function mechanisms are concurrently involved in tau-mediated pathogenesis [79]. Tau becomes hyperphosphorylated in pathologic conditions, resulting in reduced binding affinity for microtubules and disengagement from microtubules [64]. This loss of tau binding is thought to affect microtubule structure and function in neurons [45, 64, 80, 88]. The dissociation of hyperphosphorylated tau from microtubules can also hasten the formation of tau oligomers and aggregates, which are thought to provoke a gain of toxic function that negatively impacts neuronal function [65]. Pathologic tau also promotes neuroinflammation and glial activation, which may contribute to neurodegeneration and brain atrophy in the central nervous system [49]. An important aspect of tau pathology, as well as other amyloid-type brain pathologies, are recent findings suggesting that there is cell-to-cell spreading of tau pathology along neural networks. This appears to result from the release of misfolded tau from neurons harboring tau inclusions that can be internalized by interconnected neurons, thereby acting to seed tau inclusions in the recipient cell and causing the spreading of tau pathology [26].

To identify genetic modulators for tau pathology, genome-wide association studies (GWAS) have been conducted and a few potential genetic risk factors were found in the tauopathy frontotemporal dementia [9, 44, 73, 86], including ArfGAP with SH3 Domain, Ankyrin Repeat and PH Domain 1 protein (ASAP1). However, how ASAP1 and other putative risk factor genes might affect tau pathology is presently unknown, and gaining mechanistic insights could lead to new therapeutic strategies for these diseases. Reverse genetics is another strategy to identify proteins involved in the regulation of tau pathology that might serve as targets for tau therapies, and Suppressor of Tauopathy proteins (SUTs) were identified using this approach in *C. elegans* models that develop tau aggregates through overexpression of human mutant tau [29]. Nonsense mutations on two genes (*sut-1* and *sut-2*) were found to mitigate tau aggregation and neuron degeneration [29]. Importantly, loss of the mammalian ortholog of sut-2, MSUT2, was also shown to reduce insoluble tau pathology and mitigate cognitive decline in transgenic models of tauopathy [30, 82]. Although MSUT2 is a promising modulator of tau pathology in transgenic animal models with tau overexpression, little is known about the effectiveness of MSUT2 suppression in inhibiting sporadic forms of tauopathies without tau overexpression. Moreover, due to the mRNA modifying function of MSUT2 [42, 70]*,* knockout or knockdown of MSUT2 in mammalian models may reveal differentially expressed gene products that mediate the MSUT2 effect on tau pathogenesis. Thus, our objectives here were to further investigate the role of MSUT2 in models of seeded tau pathology, and to identify key MSUT2-regulated gene products that may act downstream of MSUT2.

In this regard, whereas transgenic mouse models reproduce certain critical features of tauopathy, i.e., aggregation of tau protein, behavioral and cognitive deficits, and neurodegeneration [27, 58], there are limitations to these models that are worth noting. Perhaps foremost, these models typically depend on substantial overexpression of tau that is not observed in sporadic tauopathies. In one instance, the insertion of the human *MAPT* gene in a mouse model has been shown to lead to dramatic genomic changes independent of tau pathology [24]. Notably, a MAPT knock-in model with normal levels of tau expression does not develop human-like tau aggregates [72] and transgenic mouse models also typically fail to reproduce the heterogeneity of human tau pathology seen in humans and therefore may not fully recapitulate the diversity of human disease. Finally, transgenic mouse models often do not mimic the spatiotemporal development of tau pathology in humans, which complicates investigation into the spreading of tau pathology among cell populations and brain regions.

Several studies have now demonstrated that tau aggregates from human patients can seed stereotypical tau pathology and cause neuronal dysfunction in cell and animal models in the absence of tau overexpression [28]. Furthermore, seeding these models [13, 34, 39, 56, 60, 85] with brain-derived tau from different tauopathies recapitulate aspects of the heterogeneity observed in the human diseases. Thus, these models mimic many aspects of the development of tau pathology observed in human patients in the absence of tau overexpression, allowing for investigation of the spatiotemporal development of tau pathology bearing similarity to that found in human patient brains. Here, we have further investigated the mechanisms of MSUT2-regulated tau-mediated pathogenesis using MSUT2 knock out (KO) or knockdown (KD) in combination with these previously developed in vitro and in vivo tau seeding models [28, 33, 34, 59], utilizing pathologic tau isolated from human postmortem brains or human tau seeds generated after amplification of human brain-derived tau. Finally, we have investigated changes in gene expression that result from MSUT2 knockout (KO) in an in vitro mouse primary neuron model via single-cell RNA sequencing, leading to the identification of an adenosinergic signaling pathway downstream of MSUT2 that regulates tau seeding. Moreover, our data suggest that this adenosinergic signaling acts through ASAP1, which regulates tau seed endocytosis via its influence on macropinocytosis in the tau spreading models. These studies elucidate a mechanism by which MSUT2 might regulate the spreading of tau pathology and suggest potential therapeutic strategies to reduce tau pathology.

## Materials and methods

### Study design

The overall objective of this study was to investigate the mechanism of MSUT2 on tau spreading and to explore the potential therapeutic role of its downstream factors. The study employed different mouse models (including wild-type tau spreading models, MSUT2 KO mouse, 5xFAD transgenic mouse, and 5xFAD/MSUT2 KO mouse) and subjected them to the injection of different tau strains or proteinopathy. The induced tau pathogenesis and associated protein pathologies were then analyzed using biochemical and immunohistochemical assays. Transcriptomics were performed using sc-NT-seq technique and confirmed by biochemistry, immunocytochemistry and live imaging. All experiments were conducted at the Center for Neurodegenerative Disease Research. The brain sections were imaged, and the area occupied by different immunoreactive staining of tau, Aβ plaque, or TDP-43 pathologies was quantified using software like HALO (Indica Labs) or QuPath software. Blind counts were made of the number of pathologic p-tau positive staining and quantifications were systematically performed throughout the whole mouse brain. Semi-quantitative analyses were conducted to create heat maps representing the abundance and distributions of tau pathologies throughout the brain. The sample size was determined by power analysis based on previous studies [34, 85] to gain statistical power to discern 20% of differences. All experiments were repeated with at least three biologic repeats. No data points were excluded unless specified.

### Animals

The MSUT2 KO mouse line was generated in a previous study [83]. Specifically, B6-Zc3h14tm1a mice were crossed with other mouse strains carrying the Flp and Cre recombinases, resulting in the removal of exon 13 of the MSUT2 gene and all inserted transgene sequences. The generated line was maintained on a C57BL/6 background for the study. Homozygous (MSUT2^−/−^) and wild-type littermates (MSUT2^+/+^) were compared in the degree of tau pathology (seeded by human tau seeds). A bigenic 5xFAD/MSUT2 KO mouse line was generated by crossing MSUT2 KO mice with 5xFAD mice[61]. APP^+/−^/PS1^+/−^/MSUT2^−/−^ and APP^+/−^/PS1^+/−^/MSUT2^+/+^ mice were used to compare the degree of Aβ plaque and tau pathology. The cohort included both male and female mice. All animal protocols were approved by the University of Pennsylvania's Institutional Animal Care and Use Committee (IACUC).

Primers for PCR genotyping were as follows: MSUT2 mice: MSUT2 WT_F primer 5′-GGGTTTGGGGCAGATTTATT-3′; MSUT2 WT_R 5′-CAAAGCTCCAGGGATGGTTA-3´; MSUT2 KO_F 5′-GGCAGTGTCATTTGTTGGCT-3′; MSUT2 KO_R 5′-GGACATTTCTGATCAAGGCACTG-3′. 5xFAD mice: PS1_F 5′-AATAGAGAACGGCAGGAGCA-3′, PS1_R, 5′-GCCATGAGGGCACTAATCAT-3′; Internal ctrl-1_F 5′-CTAGGCCACAGAATTGAAAGATCT-3′, Internal ctrl-1_R 5′-GTAGGTGGAAATTCTAGCATCATCC-3′; APP_F 5′-AGGACTGACCACTCGACCAG-3′, APP_R 5′-CGGGGGTCTAGTTCTGCAT-3′; Internal ctrl-2_F 5′-CAAATGTTGCTTGTCTGGTG-3′, Internal ctrl-2_R 5′-GTCAGTCGAGTGCAC AGTTT-3′.

### Human tau seed enrichment

All human cases from the Center for Neurodegenerative Disease Research (CNDR) brain bank containing AD, CBD and PSP-tau were selected and prescreened to exclude comorbidities of Lewy body and TDP-43 pathologies based on immunohistochemical staining [71]. The fronto-cortical regions of patient brains were used to sequentially extract AD, CBD, and PSP-tau as previously described [85]. Specifically, brain homogenate was prepared in 9 volumes (v/w, ml/g) of PHF buffer (10 mM Tris, 10% sucrose, 0.8 M NaCl, 1 mM EDTA, pH 7.4) with 0.1% sarkosyl, proteinase inhibitor and phosphatase inhibitor in a glass homogenizer and spun at 10,000*g* for 10 min at 4 °C following homogenization. The supernatant (sup 1) was collected, sarkosyl was added at 1% of the final concentration, and the solution was incubated for 1.5 h at RT in a beaker with stirring, followed by a 150,000*g* spin for 75 min at 4 °C. The pellet was collected, briefly washed with PBS to clean the myelin, and resuspended in PBS (pel 1). Pel 1 was then spun at 150,000*g* for 75 min to remove the sarkosyl, and the resulting pellet was collected as pel 2. The pel 2 from AD cases was resuspended in PBS, thoroughly sonicated, spun at 10,000*g* for 10 min at 4 °C, and the supernatant was collected to be used as enriched tau seeds. Conversely due to the relatively lower tau burden in the tissue of PSP and CBD cases, the collected pel 2 was used for tau seeding.

### Immunoblots

Hippocampi were dissected from the collected brains and homogenized in nine volumes of PHF extraction buffer containing 1% Triton-X100. Following homogenization, the resulting lysate was spun at 100,000*g* for 30 min at 4 °C. The supernatant was collected as the sarkosyl-soluble fraction (Sark-S) and the pellet saved as the sarkosyl-insoluble fraction (Sark-P). The immunoblots were loaded with 15 μg of total proteins from TX-S and Sark-S. Sark-P was loaded at a ratio of 10:1 to the respective TX-S fractions. Primary neurons were washed once with PBS and cell lysate was harvested in PBS containing 1% sarkosyl and proteinase inhibitor cocktail. The lysate was incubated on ice for 15 min before being spun at 100,000 g for 30 min at 4 °C. The resulting supernatant was kept as the soluble fraction and the pellet was suspended in PBS as the insoluble fraction. Immunoblots were generated as described previously [85]. Primary antibodies were incubated at 4 °C overnight, and secondary antibodies were incubated for 2 h at room temperature. Photomicrographs were taken using an immunoblot imager (Li-Cor Biosciences). Optical densities were measured using the Imagestudio^®^ Software.

### Immunohistochemistry and immunofluorescence staining

Mouse brains were perfused with PBS at a flow rate of 2 mL/min for 15 min and immersion fixed within 10% neutral buffered formalin (NBF) following a previously described protocol [85]. Six-μm thick sections were cut and used for the immuno-staining. For immunohistochemistry, several primary antibodies were used to stain brain sections and development was performed via a polymer horseradish peroxidase detection kit (Biogenex). For immunofluorescence, brain sections were incubated with primary antibodies overnight at 4 °C followed by a 2 h incubation of Alexa Fluor-conjugated secondary antibodies (Thermo Fisher Scientific). A 0.3% Sudan black solution for 1 min was used to quench auto-fluorescence. Quantification was performed within the region of interest (ROI) using threshold-based classifier by QuPath software. All the conditions to be compared were quantified using the same classifier. For IHC, 4–5 consecutive sections were quantified for hippocampus and entorhinal cortex.

### Stereotactic injection

Mice were brought to a surgical plane of anesthesia via a cocktail drug mix (KAX) consisting of ketamine–xylazine–acepromazine, and were immobilized in a stereotaxic frame (David Kopf Instruments) before being aseptically inoculated with human brain extracts or synthetic mouse α-synuclein preformed fibrils in the dorsal hippocampus and overlying cortex of one hemisphere (bregma − 2.5 mm; lateral 2 mm; depth − 2.4 mm and − 1.4 mm from the skull) using a Hamilton syringe as described previously [85]. Injected tau seeds were prediluted into concentrations of 0.4 µg tau/µL for AD-tau, 0.25 µg tau/µL for CBD-tau, and 40 ng tau/µL for PSP-tau. Each of the two injection sites received 2.5 μl of inoculum and materials were injected into the hippocampus first (− 2.4 mm from the skull) before the needle was pulled vertically upwards to the cortical injection site (− 1.4 mm from the skull). Mouse body temperature was maintained at 37 °C using a heating pad during and after surgery until mice were awake under a heating lamp for 2 h.

### Connectome correlation of tau pathology

Blind selection of slides, annotation, and quantification was performed by a research assistant with no knowledge of the mouse genotype. Coronal mouse brain sections were selected approximately every 20 slides, and selected slides were scanned before scanned files were imported into the image analysis by QuPath software, for annotation and quantification based on the Allen brain atlas. One hundred seventy two annotations (per mouse) were generated on coronal sections based on best fitting stereotypical regions at 2.10 mm, 0.98 mm, − 1.22 mm, − 2.92 mm and − 4.48 mm relative to bregma, as laid out in a previously published study [15, 85]. Annotation of regions on each coronal section was performed manually and was followed by a manual cleaning process to reduce areas subject to edge effects or areas that contained significant background staining. The same classifier was applied to all of the stereotypical regions. The anterograde and retrograde connectivity strength of the quantified areas to the injection sites was determined using a previously published study [62].

### Heatmaps of tau pathology

Heatmaps were generated for the distribution of AT-8 tau pathology as previously described [28]. Scores were given in each region of the brain for the presence of tau pathology between 0 and 3, with 0 representing complete lack of tau pathology and 3 representing the most abundant tau pathology. The semi-quantitative scoring of AT-8 tau pathology was performed in a blind manner.

### Primary neuron

CD1 mouse cortices and hippocampi were dissected at embryo day 16–18 and dissociated with papain (Worthington Biochemical Corporation). Neurons were resuspended in neural basal medium (Gibco, 21,103) with 2% B27 (Gibco), 1 × Glutamax (Gibco), and 1 × Penicillin/Streptomycin (Gibco). Coverslips or plates were coated with poly-d-lysine (0.1 mg/ml, Sigma-Aldrich) overnight in borate buffer (0.05 M boric acid, pH 8.5) at room temperature. Cells were plated at a density of 50,000 cells/cm^2^ for all types of plates, and 5% FBS was added to the cell suspension when plating. Neural basal medium without FBS was used to replace plating medium at day 1 in vitro (DIV1).

### Antisense oligonucleotides, compounds, and tau seeds treatment

Antisense oligonucleotides were obtained from AUM BioTech, LLC. ASOs and pharmaceutical compounds were diluted in culture medium before being added to neurons at up to the proper concentration for DIV2 (ASOs) or DIV5 (compounds). At DIV7, medium was replaced with a conditioned medium that contained a 1:1 ratio of old and fresh medium. Neural basal medium was used to predilute tau seeds before the seeds were sonicated using a bath sonicator. Tau seeds were then added at a dose of 50 ng tau/well (96wellplate format) to neurons and allowed to incubate with the neurons for 14 days until DIV 21 to induce tau pathology. All the ASOs were treated with 1 μM end concentration unless specified.

### Immunocytochemistry

Cells were washed with PBS in a plate washer (BIOTEK) four times at DIV 21, extracted for 10 min with 1% hexadecyltrimethylammonium bromide (Sigma-Aldrich) for 10 min following fixation in PBS with sucrose and 4% paraformaldehyde for 10 min. Fixed cells were then incubated overnight with primary antibodies at 4 °C and for 2 h with Alexa Fluor-conjugated secondary antibodies (Thermo Fisher Scientific) at room temperature. The InCell scanner was used to obtain Photomicrographs which were quantified using the InCell developer toolbox software (1.9.2) as described previously [28].

### Fluorescent labeling of recombinant protein

T40 tau monomers were expressed in BL21 (DE3) RIL E. coli. Cells, and were later purified using fast protein liquid chromatography based on previous reports [28]. Nanodrop 1000 (ND-1000, Spectrophotometer) was used to determine the concentration of recombinant tau. Succinimidyl ester based HiLyte fluor 488 microscale protein labeling kit (AnaTag) or pHrodo™ Red Labeling Kit (Thermo Fisher) was used to fluorescently label purified monomers. BODIPY maleimide dye was used to label prepared α-syn monomer as previously described [32]. Nanodrop 1000 was used to test labeling efficiency depends on the absorbance of different dyes. For fibrillization, to avoid the self-quenching of fluorophores after fibrilization, monomeric proteins were used that contained a mixture of 20% of fluorescently labeled and 80% unlabeled monomers.

### Generation of fluorescently labeled tau seeds and mouse α-syn pffs

Tau seeds were generated with a slightly modified protocol [85]. Specifically, a thermocycler was used to apply heat treatment to enriched tau seeds at 56 °C for 30 min. Following heat treatment, a bath sonicator was used to sonicate tau seeds in sizes for 20 min, with 30 s of sonication followed by 30 s of rest at the highest intensity. Tau seeds were then mixed with T40 monomer for a total tau concentration of 40 µM (4 µM tau seeds, 36 µM monomeric tau). All the reactions were conducted in PCR tubes containing sterile phosphate-buffered saline with 50 µl volume on a thermomixer for 10 days at 37 °C. On day 0 and day 10 samples were collected and kept frozen in a − 80 °C freezer until use. Samples were spun for 30 min at 100,000*g* with an ultracentrifuge (Optima, Beckman Coulter) to remove unreactive monomeric tau at the end of the reaction. The pellet was resuspended in PBS after removal of the supernatant, and tau concentration was estimated using immunoblots. Only pellet fractions were used in the live imaging experiments for uptake assays of pathogenic tau seeds.

Mouse α-synuclein (α-syn) PFFs were generated using a previously established protocol [41]. In brief, monomers of wild-type mouse α-synuclein were purified using FPLC and subsequently labeled with bodipy dye, following the method described in an earlier publication [41]. The labeled monomers were then subjected to agitation at 5 mg/ml, 1000 rpm, and 37 °C for 5 days to facilitate the formation of PFFs. The resulting PFFs were diluted with PBS and subsequently applied to primary neuron cultures at the specified dosage.

### Sedimentation assay

A revised version of the previously used sedimentation assay [85] was conducted by mixing 1 µl of the sample with 19 µl of PBS (0.1% sarkosyl, *w*/*v*) before being spun for 30 min at 100,000*g* with an ultracentrifuge (Optima, Beckman Coulter). The supernatant was then carefully removed, before the pellet was resuspended in 20 µl of PBS. The loading buffer was mixed with the fractions for use in immunoblots.

### Live cell imaging

Neurons were treated with ASOs in a µ-Dish 35 mm chamber (ibiTreat, Thermo fisher scientific) at DIV2 or with compounds at DIV5 followed by treatment with fluorescently labeled tau seeds at a dose of 200 ng/quadrant at DIV7. Extracellular signals of pH-insensitive dyes (FTIC channel) were quenched through addition of 500 µM of trypan blue to the neurons before imaging. A live imaging microscope was used to take images at 37 °C and 5% CO_2_ conditions within a closed, humidified chamber.

### 4sU-labeling of neuronal cell culture

KO and WT primary neurons were prepared as previously described. Cells were maintained at 37 °C with 5% CO_2_ for 10 days (DIV10). FoursU (Alfa Aesar, J60679) was dissolved in dimethylsulfoxide to make 1 M stock and added to the neuron culture at a final concentration of 100 μM. After 4 h of labeling, cells were rinsed once with PBS before being dissociated into single-cell suspensions containing TrypLE-Express (Gibco, 12,605,010) for 5 min at 37 °C. Cells were then counted with hemacytometer and fixed with 80% methanol as described [67]. Samples were stored at -80C until library preparation.

### scNT-seq library prep and sequencing

A resuspension buffer (0.01% BSA in DPBS with 0.5% RNase-inhibitor) was used to rehydrated methanol fixed samples, which were then counted with Countess II (Life Technologies, AMQAF1000). Cells were diluted to 100 cell/μl in resuspension buffer and loaded to a Drop-seq microfluidic device. A droplet microfluidics-based cell and barcoded bead co-encapsulation library preparation was performed as previously described following minor modifications (on-beads Bst3-based Second-strand synthesis reaction after RT and exonuclease I treatment were included) [67]. See open access protocol for more details: Protocol Exchange https://doi.org/10.21203/rs.3.pex-1019/v1 (2020). Libraries were quantified via Qubit 3.0 (Invitrogen). After library quality was determined via Bioanalyzer (Agilent)sequencing was performed on an Illumina NextSeq 500 using the 75-cycle High Output v2 kit (Illumina). The library was then loaded at 2.0 pM and Custom Read1 Primer (GCCTGTCCGCGGAAGCAGTGGTATCAACGCAGAGTAC) was added at 0.3 μM to position 7 of the reagent cartridges. The sequencing configuration was 20 bp (Read 1), 8 bp (Index 1) and 60 bp (Read 2).

### Read alignment, cell-type clustering, and identification of differentially expressed genes

Paired-end sequencing reads of scNT-seq were processed as previously described [67]. Each cell barcode tagged mRNA read (Read 2) was trimmed of sequencing adaptors and poly-A sequences, and aligned to the mouse reference genome (mm10, Gencode release vM13) using STAR v2.7.2b. Both exonic and intronic reads that mapped to predicted strands of annotated genes were retained for the downstream analysis. The raw digital expression matrices were generated with the Drop-seq Tools v1.12 software and loaded into the R package Seurat (v 4.1.0). Only genes detected in > 10 cells were retained [53]. Cells with fewer than 300 or more than 6000 detected genes were removed. Cells with a relatively high percentage of UMIs mapped to mitochondrial genes (≥ 10%) were also discarded.

After removing low-quality cells, 4855 and 2624 cells from wild-type and KO samples were retained. For normalization, UMI counts for all cells were scaled by library size (total UMI counts), multiplied by 10,000 and transformed to log space. The top 3000 HVGs were identified using the function FindVariableFeatures with the vst method. The expression level of HVGs in the cells was scaled and centered for each gene across cells and was subjected to PCA. Harmony was then used to adjust the principal components for batch effects with default parameter [43]. The most significant 40 PCs were selected and used for two-dimensional reduction by UMAP in Seurat with the default parameters. Clusters were identified using the function FindCluster in Seurat with the resolution parameter set to 0.3. Major cell types were annotated based on the expression of marker genes (Ex: *Neurod2*, *Neurond6*; Inh: *Dlx1*, *Gad1*, *Gad2*; OPC: *Pdgfra*, *Olig2*; Astro_RG: *Aldh1l1*, *Aldoc*; MG: *C1qc*, *C1qb*). For each major cell type, differential gene expression was computed between genotypes (WT vs. KO) using Wilcoxon rank sum test (using the function FindMarkers in Seurat).

### Estimation of RNA biogenesis rate and degradation rate constant

To quantify the newly synthesis RNA, we applied the newly developed *dynast* (v 1.0.1, https://github.com/aristoteleo/dynast-release), an inclusive and efficient command-line toolkit for preprocessing data from metabolic labeling-based scRNA-seq experiments. Fastq file from each sample were subjected to standard *dynast* runs (align, consensus, count), then the fraction of labeled RNA was estimated with the dynast estimate command (–method alpha). To estimate RNA biogenesis rate and degradation rate of excitatory neurons (Ex), the dynast output (h5ad file) from each sample was loaded into *dynamo* (v 1.1.0, https://dynamo-release.readthedocs.io/en/latest/) [68]. The excitatory neurons were based on the annotation of the Seurat project and were subjected to the dynamics function (one_shot_method = “sci_fate”, model = “deterministic”). The RNA biogenesis rate and degradation rate of differential expression genes were filtered (non-zero values were kept) before visualized with heat-map.

### Data analysis and statistics”

Unpaired t-tests were performed for comparisons of twoexperimental groups. For comparisons involving more than two groups, one-way ANOVA and two-way ANOVA tests were performed, followed by Tukey post hoc multiple comparison testing if not specified. Prism software (GraphPad Software, Inc) was used to perform all statistical testing. Statistical significance was determined as *p* < 0.05. Unless specified otherwise, data was presented in the format of mean ± standard deviation.

## Results

### Neuronal MSUT2 level is associated with the presence of tau pathology in human brains

**Fig. 1:**
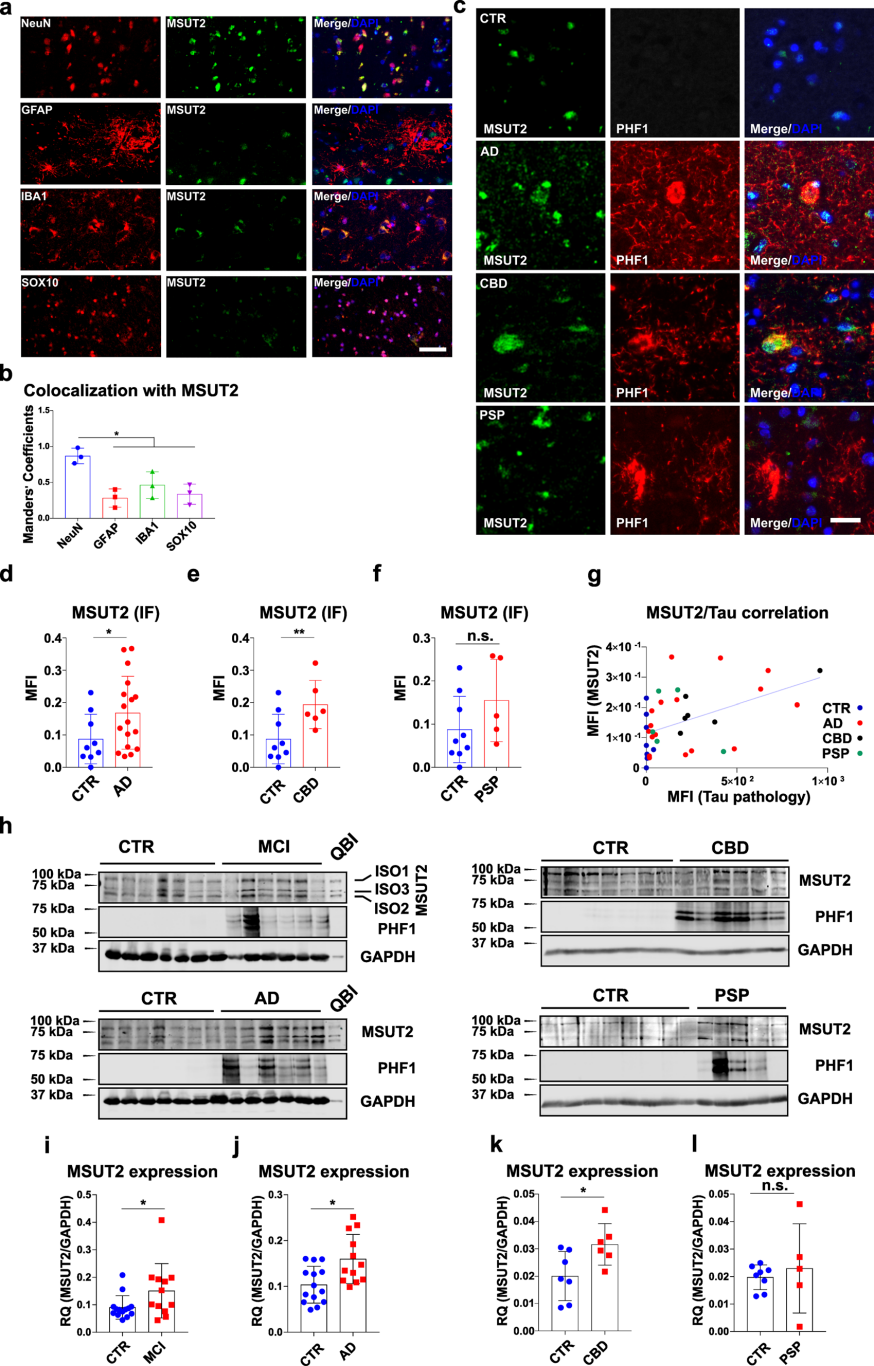
MSUT2 expression is associated with human tau pathology. **a** Immunofluorescence co-staining of frontal cortical human brain sections with antibodies against neurons (NeuN), astrocytes (GFAP), microglia (IBA1), oligodendrocytes (SOX10) and MSUT2. Scale bar = 50 µm. **b** Measurement of MSUT2 and cellular fluorescent signal co-localization in **a**. *P < 0.05 by one-way ANOVA followed by Tukey’s post hoc test, *n* = 3. The error bars represent the standard deviation. **c** Representative images of non-tauopathy control (CTR), Alzheimer’s disease (AD), Corticobasal degeneration (CBD), and Progressive supranuclear palsy (PSP) patient brain sections (frontal cortex) co-stained with MSUT2 and p-tau (PHF1) antibodies to reveal MSUT2 protein and tau pathology. Scale bar = 5 µm. **d**–**f** Quantification of MSUT2 immunoreactivity (MFI = mean fluorescence intensity) in non-tauopathy control (CTR) vs. diseased brain sections as exemplified in **c**. MSUT2 area is normalized to the neuron counts. Four random images of the cortical region were used for the quantification of each case. *P < 0.05, **P < 0.01, n.s., not significant, by t-test when comparing CTR (*n* = 9) vs. AD (*n* = 17), CBD (*n* = 6), or PSP (*n* = 5). The error bars represent the standard deviation. **g** Correlation of MSUT2 expression and tau pathology (PHF1 immunoreactivity) quantified from sections as exemplified in **c**. *R*^2^ = 0.2256, *P* = 0.0030 by normal linear regression. Four random images of the cortical region/case were used for the quantification of both MSUT2 and PHF1. Each dot represents the mean value from one individual case. Both MSUT2 and tau pathology level was presented by the integrated mean fluorescence intensity of PHF1 staining. **h** Representative immunoblots of MSUT2 and p-tau levels in human brains and cellular homogenates probed with MSUT2, PHF1, and GAPDH antibodies. Samples include CTR, MCI (mild cognitive impairment), AD, CBD, and PSP brains. The CTR, MCI, and AD cases were separated and run on separate blots due to lane limitations. In addition, for the gels involving CTR cases in CBD and PSP, the control (CTR) cases were randomly selected from within the same CTR group. Three isoforms of MSUT2 are indicated using QBI HEK-293 cell lysate samples. **i**–**l** Quantification of MSUT2 immunoreactivity in immunoblot samples as depicted in **h**. All samples were normalized to GAPDH to gain the relative quantity (RQ). All three isoforms were included for quantification. n.s., not significant, *P < 0.05, **P < 0.01 by *t*-test, *n* = 14 CTR vs. 12 MCI, *n* = 14 CTR vs. 12 AD, *n* = 7 CTR vs. 6 CBD, *n* = 8 CTR vs. 5 PSP. The error bars represent the standard deviation

The human brain contains multiple cell populations, which are differentially affected in human tauopathies [12]. We used double immunofluorescence to co-stain human fronto-cortical sections with antibodies to MSUT2 and neuronal (NeuN), astrocytic (GFAP), microglial (IBA1), or oligodendroglial (SOX10) markers to determine MSUT2 expression in different cell types in the central nervous system. Generally, MSUT2 was found to be expressed in all types of brain cells (Fig. 1a) with a higher level of co-localization in neurons compared to the other cell types (Fig. 1b), emphasizing the potential importance of MSUT2 in neuronal populations. A previous study showed a biphasic pattern of MSUT2 expression in AD patient brains [82], in which some of the AD cases contained higher levels of MSUT2 than non-AD control brains while other AD cases contained very low levels of MSUT2. We hypothesize that this was due to variability in neuron loss in these cases. Thus, to determine if tau pathologies affects MSUT2 levels in patient brains, we measured neuronal MSUT2 in both non-tauopathy (non-tau pathology control: Parkinson’s disease, Multiple System Atrophy and non-pathologic cases) and tauopathy patients (Table 1), and determined whether there was a correlation with the amount of tau pathology in the fronto-cortical region (Fig. 1c). Our results showed that MSUT2 (in green) only occasionally co-localized with PHF-1-positive neurofibrillary tangles (NFTs, in red) in AD and CBD brains, and in general we did not observe a high level of colocalization between MSUT2-positive neurons and the presence of NFTs in diseased brains. However, the quantification of total MSUT2 and tau pathology showed that neuronal MSUT2 levels were significantly elevated in AD and corticobasal degeneration (CBD) cases (Fig. 1d, e), with a non-significant trend toward increase in PSP cases (Fig. 1f) that showed higher variability and a lower level of tau pathology than AD or CBD cases. In addition, neuronal MSUT2 positively and weakly correlated with the total amount of tau pathology (Fig. 1g) but not with patient age, disease duration, or Aβ plaque load (Fig. S1a–c).

**Table 1:** Demographic features of used human patient cases

Name	Brain atrophy	Clinical diagnosis	Neuropathological diagnosis	Braak stage	Tau burden score	Age of onset (yr)	Age at death (yr)	Disease duration (yr)	Gender
Ctr	None/normal	Normal	Alzheimer’s disease	I	0	n.a	68	n.a	Female
Ctr	None/normal	Normal	Alzheimer’s disease	0	0	n.a	65	n.a	Female
Ctr	None/normal	Normal	Pathologic aging	0	0	n.a	42	n.a	Male
Ctr	None/normal	Normal	Cerebrovascular disease	0	0	n.a	57	n.a	Male
Ctr	None/normal	Normal	Unremarkable adult brain	0	0	n.a	70	n.a	Male
Ctr	Mild	Normal	Unremarkable adult brain	0	0	n.a	66	n.a	Male
Ctr	None/normal	Normal	Alzheimer’s disease	0	0	n.a	75	n.a	Female
Ctr	None/normal	Normal	Primary age-related tauopathy	0	0	n.a	72	n.a	Female
Ctr	None/normal	Normal	Primary age-related tauopathy	I	0	n.a	56	n.a	Female
Ctr	None/normal	Normal	Primary age-related tauopathy	I	0	n.a	83	n.a	Female
Ctr	None/normal	Normal	Primary age-related tauopathy	0	0	n.a	61	n.a	Female
Ctr	Moderate	Normal	Primary age-related tauopathy	0	0	n.a	68	n.a	Female
Ctr	None/normal	Normal	Primary age-related tauopathy	I	0	n.a	62	n.a	Male
Ctr	None/normal	Normal	Primary age-related tauopathy	I	0	n.a	59	n.a	Male
CBD	Severe	FTLD-PPA (PNFA)	Corticobasal degeneration	0	3 +	66	76	10	Female
CBD	Moderate	FTLD-NOS	Corticobasal degeneration	II	3 +	39	44	5	Male
CBD	Moderate	FTLD-bvFTD	Corticobasal degeneration	II	3 +	49	52	3	Male
CBD	Severe	Corticobasal syndrome	Corticobasal degeneration	II	3 +	59	63	4	Male
CBD	Moderate	Probable Alzheimer's Disease	Corticobasal degeneration	0	3 +	50	56	6	Male
CBD	Mild	Progressive supranuclear palsy	Corticobasal degeneration	0	3 +	54	59	5	Female
PSP	Mild	Corticobasal syndrome	Progressive supranuclear palsy	II	1 +	55	65	10	Male
PSP	Mild	Progressive supranuclear palsy	Progressive supranuclear palsy	I	1 +	67	72	5	Female
PSP	None/normal	Progressive supranuclear palsy	Progressive supranuclear palsy	II	2 +	76	78	2	Male
PSP	Moderate	FTLD-PPA (PNFA)	Progressive supranuclear palsy	0	2 +	67	72	5	Male
PSP	Severe	Progressive supranuclear palsy	Progressive supranuclear palsy	II	3 +	58	63	5	Female
AD	Severe	Possible Alzheimer’s Disease	Alzheimer’s disease	VI	3 +	52	55	3	Male
AD	Mild	Probable Alzheimer’s Disease	Alzheimer’s disease	VI	3 +	79	90	11	Female
AD	Moderate	Probable Alzheimer’s Disease	Alzheimer’s disease	VI	3 +	71	82	11	Male
AD	Severe	FTLD-NOS	Alzheimer’s disease	VI	3 +	55	75	20	Male
AD	Moderate	Probable Alzheimer’s Disease	Alzheimer’s disease	VI	3 +	58	66	8	Male
AD	Moderate	Probable Alzheimer’s Disease	Alzheimer’s disease	VI	3 +	60	68	8	Female
AD	Severe	FTLD-bvFTD	Alzheimer’s disease	VI	3 +	52	62	10	Male
AD	Moderate	Probable Alzheimer’s Disease	Alzheimer’s disease	VI	3 +	50	59	9	Female
AD	Moderate	Probable Alzheimer’s Disease	Alzheimer’s disease	VI	3 +	61	70	9	Male
AD	Severe	Probable Alzheimer’s Disease	Alzheimer’s disease	VI	3 +	47	55	8	Female
AD	Moderate	Probable Alzheimer’s Disease	Alzheimer’s disease	VI	3 +	71	82	11	Female
AD	Severe	Probable Alzheimer’s Disease	Alzheimer’s disease	VI	3 +	55	66	11	Male
MCI	Severe	Mild cognitive impairment	Alzheimer’s disease	VI	2 +	66	81	15	Female
MCI	Mild	Mild cognitive impairment	Alzheimer’s disease	V	1 +	79	88	9	Female
MCI	Moderate	Mild cognitive impairment	Alzheimer’s disease	V	1 +	n.a	88	n.a	Male
MCI	Severe	Mild cognitive impairment	Alzheimer’s disease	VI	1 +	81	96	15	Female
MCI	Severe	Mild cognitive impairment	Alzheimer's disease	VI	2 +	81	92	11	Female
MCI	Moderate	Mild cognitive impairment	Alzheimer’s disease	VI	3 +	68	78	10	Male
MCI	Moderate	Mild cognitive impairment	Alzheimer’s disease	V	3 +	80	85	5	Female
MCI	Mild	Mild cognitive impairment	Alzheimer’s disease	V	2 +	74	82	8	Male
MCI	Severe	Mild cognitive impairment	Alzheimer’s disease	VI	3 +	60	72	12	Female
MCI	Mild	Mild cognitive impairment	Alzheimer’s disease	VI	1 +	72	79	7	Male
MCI	None/normal	Mild cognitive impairment	Alzheimer’s disease	VI	1 +	69	74	5	Female
MCI	Moderate	Mild cognitive impairment	Alzheimer’s disease	V	2 +	74	77	3	Male

*Ctr* non-tauopathy control, *FTLD-PPA* Frontotemporal lobar degeneration with primary progressive aphasia, *FTLD-NOS* Frontotemporal lobar degeneration-not otherwise specified, *FTLD-bvFTD* Frontotemporal lobar degeneration-behavioral-variant frontotemporal dementia, *n.a.* not available

To extend these immunohistochemical observations, we measured MSUT2 expression levels in human tauopathy cases using immunoblots (Fig. 1 h). The results are consistent with the immunofluorescent experiments and verified that the MSUT2 protein level was increased in patient brains with tau pathology when compared with the non-tauopathy controls (CTR). Notably, increased levels of MSUT2 could be demonstrated in different tauopathies and at different disease stages, as patients with mild cognitive impairment (MCI), AD, and CBD all showed significantly greater levels of MSUT2 (Fig. 1h–k). However, we observed no statistical difference in MSUT2 levels between PSP and CTR brains in the immunoblots, in keeping with the non-significant trend observed by immunofluorescence staining (Fig. 1f and l). In summary, these observations indicate that neuronal MSUT2 level is increased in MCI, AD and CBD brain, and there is a weak but positive correlation between MSUT2 levels and the amount of tau pathology.

### MSUT2 mediates tau spreading in vivo

Most human tauopathy cases are sporadic and independent of known genetic causes. Although it was previously reported that loss of MSUT2 mitigates tau pathology in tau transgenic models [30, 82], its relevance to sporadic human tau pathology is unclear and the specific molecular mechanism(s) of MSUT2 regulation of tau pathology have not been elucidated. Here, we undertook studies to determine if MSUT2 is involved in pathways controlling cell-to-cell spreading of tau. Due to transgene-driven overexpression of tau in transgenic models, it is difficult to tease apart the spatiotemporal development of tau pathology and the extent to which tau spreading contributes to the development of tau pathology. Therefore, we leveraged an established tau seeding and spreading model [28, 60], comparing MSUT2 knockout (KO) and wild-type mice [83] to quantitatively evaluate the effect of MSUT2 on the development and spreading of tau pathology independent of tau overexpression. Before initiating seeding studies, we first examined the expression levels of a series of key proteins that might be associated with tau pathology and overall neurobiology in brains of adult MSUT2 KO and wild-type mice. We did not observe any significant changes in the expression of the tested proteins, other than MSUT2 (Fig. S2). Given that MSUT2 was functionally knocked out at one of the zinc finger domains [83] in the MSUT2 KO mice, we did detect faint bands in the MSUT2 blots that likely result from incomplete degradation of the non-functional MSUT2 protein. These results indicate that levels of proteins involved in neuron viability (tau, APP, Fox-3), synaptic plasticity (synapsin, synaptophysin, PSD95), proteasomal activity (K48-polyUb, ubiquitin), and glial function (GFAP, Iba1) are not significantly altered in the brains of MSUT2 KO mice compared to their wild-type littermates. These findings are in keeping with the observation that MSUT2 KO mice appear to be free of significant developmental or central nervous system abnormalities [83].

**Fig. 2:**
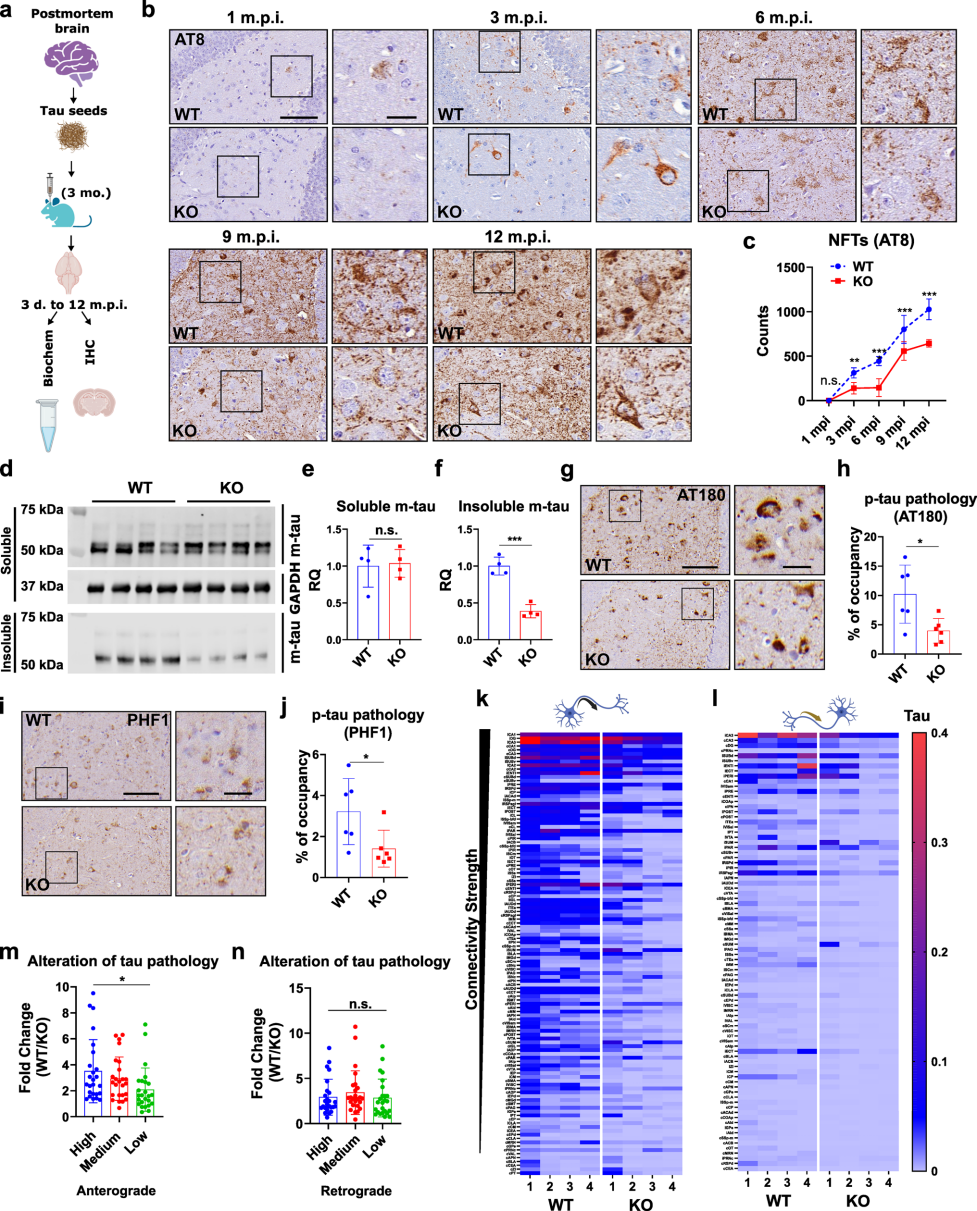
MSUT2 modulates the spatiotemporal spreading of tau pathology induced by human-derived AD-tau seeds. **a** Schematic picture of the experimental paradigm: AD-tau seeds were enriched from postmortem AD brains and stereotactically injected into the hippocampi of MSUT2 KO mice and wild-type (WT) littermates at 3 months of age. Mouse brains were collected at different time points (from 3 days to 12 months post-injection time; m.p.i.) and analyzed by immunohistochemistry and biochemistry. **b** Representative images of AD-tau-injected mouse hippocampi at 1 to 12 m.p.i. AD-tau-seeded MSUT2 KO and WT mouse tau pathology was revealed with a S199/T205-p-tau antibody (AT8). Scale bar = 60 and 15 (inset) µm. **c** Quantification of neurofibrillary tangles (NFTs) in the ipsilateral hippocampal region of the AD-tau-injected MSUT2 KO and WT mouse brains. **P < 0.01, ***P < 0.001 by two-way ANOVA followed by Bonferroni’s post hoc test, *n* = 6 per group. The error bars represent the standard deviation. **d** MSUT2 KO and WT mouse brains were injected with AD-tau and analyzed at 3 m.p.i. Proteins were extracted and fractionated from the ipsilateral hippocampi. Soluble fractions were probed with a mouse tau (m-tau) antibody T49 and GAPDH antibody as the loading control. Insoluble fractions were probed with T49 antibody. **e**, **f** Quantification of T49-positive optical density from immunoblots in **d**. Relative Quantities (RQ) were determined by normalizing T49 to GAPDH signals in the soluble fraction. n.s., not significant, ***P < 0.001 by t-test, WT vs. MSUT2 KO *n* = 4 per group. The error bars represent the standard deviation. **g** Representative images showing T231-p-tau pathology in the AD-tau-injected (12 m.p.i.) WT and MSUT2 KO mouse brains. Mouse brain sections were stained with a p-tau antibody (AT180). Scale bar = 60 and 15 (inset) µm. **h** Quantification of AT180-positive area in the ipsilateral hippocampi of the mice as depicted in **g**. *P < 0.05 by t-test, WT vs. MSUT2 KO, *n* = 6 per group. The error bars represent the standard deviation. **i** Representative images showing S396/S404-p-tau pathology in the AD-tau-injected (12 m.p.i.) WT and MSUT2 KO mouse brains. Mouse brain sections were stained with a p-tau antibody (PHF1). Scale bar = 60 and 15 (inset) µm. **j** Quantification of PHF1-positive area in the ipsilateral hippocampi of the mice as depicted in **i**. *P < 0.05 by t-test, WT vs. MSUT2 KO, *n* = 6 per group. The error bars represent the standard deviation. **k** Heatmap of tau pathology at 12 m.p.i. in different brain regions of MSUT2 KO and WT mice aligned in high (top) to low (bottom) anterograde connectivity strength to the injection site. Color hue indicates the abundance of tau pathology (Tau). *n* = 4 per group. **l** Heatmap of tau pathology at 12 m.p.i. in different brain regions of MSUT2 KO and WT mice aligned in high (top) to low (bottom) retrograde connectivity strength to the injection site. Color hue indicates the abundance of tau pathology (Tau). *n* = 4 per group. **m**, **n** Fold-change (WT/KO) in tau pathology based on different levels of anterograde and retrograde connectivity strength. High = 1st–25th ranked regions, Medium = 26th–50th ranked regions, Low = 89th–113th ranked regions (lowest 25 regions). n.s., not significant, *P < 0.05 by one-way ANOVA followed by Tukey’s post hoc test, high vs. medium vs. low connectivity region, *n* = 25 per connectivity category. The error bars represent the standard deviation

To investigate whether MSUT2 KO affects tau spreading, we enriched pathogenic tau seeds from AD postmortem brains (AD-tau) and injected them into the hippocampi of MSUT2 KO and wild-type mice at 3 months of age. We measured the turnover of tau seeds and the spreading of tau pathology at different time points post-injection (Fig. 2a). Since prior studies [2, 28] have already documented that human tau seeds can be degraded within 7–14 days post-injection, we first evaluated if the elimination of injected human tau is affected by MSUT2 genotype. To this end, we probed for AD-tau seeds at 3- and 7-days post-injection (d.p.i.) using an anti-phospho-tau (p-tau) antibody (AT8) (Fig. S3a and b) or anti-human tau antibody (HT7) (Fig. S3c and d). After quantification of the immunoreactivity of both antibodies, we found no difference between human tau injected into MSUT2 KO mice or their wild-type littermates, suggesting that the global removal of pathogenic tau seeds was not substantially affected by the loss of MSUT2 in the mouse brain.

Acute neuroinflammation may impact the viability of neurons in various types of neurodegenerative diseases [31, 49]. To evaluate if KO of MSUT2 alters markers of astrocyte or microglia activation after AD-tau seeding, we used immunohistology to assess astrocytes (GFAP) and microglia (Iba1) in the area of the injection site where glial activation can be observed in 7 d.p.i. mouse brains. Notably, no differences were observed in the area occupied by these glial cells, or in overall glial morphology, between MSUT2 KO mice and wild-type mice (Fig. S3e–h). The results suggest that MSUT2 does not affect the acute neuroinflammatory response caused by the injection of AD-tau in mouse brains. As expected, we did not observe any mouse tau pathology at 3 and 7 d.p.i. in AD-tau injected mouse brains (Fig. S3 a and b). However, the AD-tau injected mice began to develop mouse tau pathology at 1-month post-injection (m.p.i.), which could be revealed with the anti-pTau AT8 antibody (Fig. 2b). As in human AD patient brains, the induced-AD-tau pathology in the mouse brain is present as both neurofibrillary tangles and neuritic tau pathology, which likely represent different stages of tau spreading [26]. We compared both forms of tau pathology by quantifying the number of AT8-positive NFTs, the area occupancy of AT8-positive staining, and the ratio of NFTs to total area of tau pathology after AD-tau injection in MSUT2 KO mice and their wild-type littermates. Our results show that NFTs are significantly reduced in MSUT2 KO mice (Fig. 2c) up to 12 m.p.i. Quantification of total tau pathology (combined NFT and neuritic tau pathology) shows a similar trend of significant decrease in the MSUT2 mouse brains (Fig. S4a).To further confirm alteration in pathologic tau in the MSUT2 KO mice, we analyzed the insoluble mouse tau aggregates in 3 m.p.i brains and found that the amount of sarkosyl-insoluble tau was decreased in the MSUT2 KO mouse brain (Fig. 2d and f), while the soluble tau remained unchanged (Fig. 2d and e). Moreover, we confirmed the decrease in tau pathology in the MSUT2 KO mouse brains with two additional anti-p-tau antibodies, AT180 (Fig. 2g and h) and PHF1 (Fig. 2i and j). We also evaluated the astrocytic and microglial response during the progression of tau spreading and found no difference between MSUT2 KO and wild-type littermate brains (Fig. S4b and c), which further implicates a neuronal role of MSUT2 in tau pathology in these models. Our data suggest that MSUT2 regulates temporal tau seeding and/or spread in vivo.

We next determined how MSUT2 expression affects the spatial distribution of tau pathology at the neuronal connectome level. Assuming the initial seeding of tau pathology started near the injection sites of the mouse brains [15, 19, 57], we compared the distribution of AT8-positive tau pathology in 12 m.p.i mice and correlated the severity of tau pathology burden with neuronal connectivity strength to the injection sites [62]. At this time point, tau pathology was present in most brain regions. We quantitatively measured tau pathology in more than 100 regions of the brain and analyzed the abundance of tau pathology based on anterograde and retrograde connectivity of neurons with the injection sites. Initially, we noticed that tau pathology in some brain regions is differentially affected by KO of MSUT2, with certain brain regions having higher (e.g., dentate gyrus) or lower (e.g., midbrain reticular nucleus) pathology relative to wild-type mice (Fig. S5a). When we aligned brain regions containing tau pathology with the connectivity strength, we found the distribution of tau pathology was well correlated with the anterograde connectivity strength (Fig. 2k) and to a lesser extent, with retrograde connectivity strength (Fig. 2l, Data 1) in both MSUT2 KO and wild-type mice. Furthermore, when comparing wild-type mice with MSUT2 KO mice, we observed a global reduction in tau pathology in the latter, which was again more significant in regions anterogradely connected to the injection site compared to regions retrogradely connected to the injection site. The fold-change in tau pathology (WT/KO) was significantly higher in the anterogradely connected regions with high connectivity strength than those ones with low connectivity strength (Fig. 2m). This trend was not observed in the retrogradely connected regions, perhaps due to there being less overall tau pathology in retrogradely than in anterogradely connected neurons (Fig. 2n). A distribution heat-map of tau pathology using a semi-quantitative scale (Fig. S5b) shows that the brain regions containing tau pathology had lesser pathologic burden in the MSUT2 KO mice at earlier time points (3, 6, 9 m.p.i.). Altogether, our observations suggest that loss of MSUT2 reduces the neuron connectome-dependent spreading of tau pathology throughout the brain, perhaps by inhibiting the seeding of tau pathology.

### MSUT2 specifically modulates tau pathology but not other proteinopathies

**Fig. 3:**
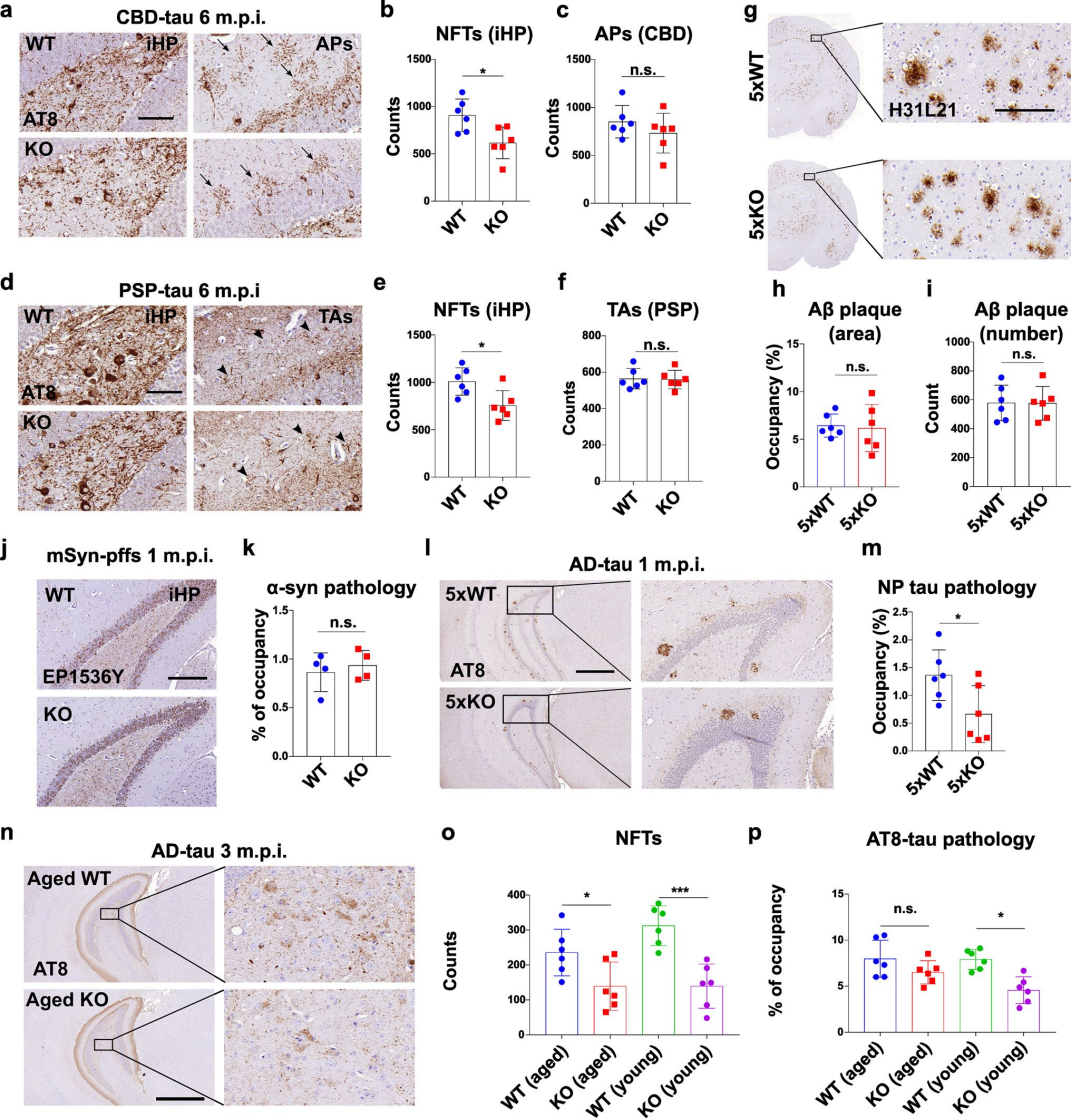
MSUT2 modulates tau pathology but not Aβ or α-synuclein pathology. **a** Representative images of AT8-positive tau pathology in CBD-tau-injected MSUT2 KO (KO) and wild-type (WT) mouse brains. Mice were injected with CBD-tau at 3 months of age and brains were collected, sectioned, and stained with AT8 antibody for tau pathology at 6 m.p.i. iHP = ipsilateral hippocampus; NFTs = neurofibrillary tangles; APs = astrocytic plaques (arrows). Scale bar = 50 μm. **b**, **c** Quantification of NFTs and APs (count) in sections stained as in **a**. *P < 0.05, n.s., not significant by t-test, MSUT2 KO (KO) vs. wild-type (WT) mouse, *n* = 6 mice per group. Error bars represent the standard deviation. **d** Representative images of AT8-positive tau pathology in PSP-tau-injected MSUT2 KO (KO) and wild-type (WT) mouse brains. Mice were injected with PSP-tau at 3 months of age and brains were collected, sectioned, and stained with AT8 antibody for tau pathology at 6 m.p.i. iHP = ipsilateral hippocampus; NFTs = neurofibrillary tangles; TAs = tufted astrocytes (arrowheads). Scale bar = 50 μm. **e**, **f** Quantification of NFTs and TAs (count) in sections stained as in **d**. *P < 0.05, n.s., not significant by t-test, MSUT2 KO (KO) vs. wild-type (WT) mouse, *n* = 6 mice per group. Error bars represent the standard deviation. **g** Representative images of Aβ plaque pathology in the brains of 5xFAD/MSUT2 KO (5xKO) and 5xFAD/MSUT2 wild-type (5xWT) mice. Mice were sacrificed at 8 months of age. Mouse brain sections were stained with H31L21 (Aβ42) antibody to reveal the Aβ plaques. Scale bar = 100 µm. **h** Quantification of Aβ plaque-positive area (H31L21 immunoreactivity) in 5xKO and 5xWT mouse brains in sections stained as in **g**. n.s., not significant by t-test, 5xFAD vs. 5xKO, *n* = 6 mice per group. Error bars represent the standard deviation. **i** Counts of Aβ plaque numbers in 5xKO and 5xWT mice in sections stained as in **g**. n.s., not significant by t-test, 5xFAD vs. 5xKO, *n* = 6 mice per group. Error bars represent the standard deviation. **j** Representative images of ipsilateral hippocampal regions of MSUT2 KO (KO) mice and wild-type (WT) littermates injected with mouse-α-synuclein preformed fibrils (mSyn-pffs) at 1 m.p.i. Mouse brain sections were stained with EP1536Y antibody to reveal α-synuclein pathology. Scale bar = 200 μm. **k** Quantification of α-synuclein pathology area (EP1536Y immunoreactivity) in the ipsilateral hippocampal regions of mSyn-pffs-injected MSUT2 KO (KO) mice and wild-type (WT) littermates in sections stained as in **j**. n.s., not significant by *t*-test, MSUT2 KO (KO) vs. wild-type (WT) mouse, *n* = 4 mice per group. Error bars represent the standard deviation. **l** 5xKO and 5xWT were injected with AD-tau at 7 months of age. Mouse brains were collected, sectioned and stained for neuritic plaque (NP) tau pathology with AT8 antibody at 1 m.p.i. representative images show ipsilateral hippocampal regions of injected mouse brains. Scale bar = 500 µm. **m** Quantification of NP tau pathology area in AD-tau-injected 5xKO and 5xWT mice at 1 m.p.i. in sections stained as in **l**. *P < 0.05 by *t*-test, 5xFAD vs. 5xKO, *n* = 6 mice per group. Error bars represent the standard deviation. **n** Brain sections from aged (12–15-month-old) AD-tau-injected MSUT2 KO (KO) and wild-type (WT) littermates at 3 m.p.i. were probed with AT8 antibody for tau pathology. Representative images show AT8-positive staining in the caudal hilus regions of the injected mouse brains. Scale bar = 500 µm. **o** Neurofibrillary tangles were counted in the ipsilateral hippocampal regions of AD-tau-injected aged MSUT2 and wild-type littermates at 3 m.p.i. in sections stained as in **n**. *P < 0.05, ***P < 0.001 by one-way ANOVA followed by Newman-Keuls’ post hoc test, MSUT2 KO (KO) vs. wild-type (WT) mouse, *n* = 6 mice per group. Error bars represent the standard deviation. **p** AT8-positive tau pathology area was quantified in the ipsilateral hippocampal regions of AD-tau-injected aged MSUT2 KO (KO) and wild-type (WT) littermates in sections stained as in **n**. n.s., not significant, *P < 0.05 by one-way ANOVA followed by Newman-Keuls’ post hoc test, WT vs. KO, n = 6 mice per group. Error bars represent the standard deviation

Human tau pathology exhibits strain-like properties, including neuronal and non-neuronal forms of tau pathology, and differing isoform composition and bioactivity of tau aggregates [34, 59, 85]. We thus wanted to evaluate whether the modulatory effects of MSUT2 KO on tau pathogenesis that was observed upon AD-tau seeding would also be seen after seeding with pathologic tau isolated from other subtypes of tauopathies. We previously established models of FTLD-tau pathology after seeding mouse brains with tau preparations enriched from human brains with CBD (CBD-tau) or PSP (PSP-tau). These mouse models recapitulated the tau isoform composition and cell-type distribution of tau pathology observed in their human counterparts [34, 85]. Here, we injected CBD-tau (Fig. 3a–c, Fig. S6a–c) and PSP-tau (Fig. 3d–f, Fig. S6d–f) into the MSUT2 KO mice and wild-type littermates and measured the amount of tau pathology that formed over time using the AT8 antibody. The results show that NFTs were reduced in multiple brain regions of MSUT2 KO mice compared to wild-type mice after injection of either CBD-tau or PSP-tau (Fig. 3b, e). To determine if loss of MSUT2 affects non-neuronal forms of tau pathology, we quantified CBD-specific astrocytic plaque tau (APs), PSP-specific tufted astrocyte tau (TAs) (Fig. 3c, f) and oligodendroglia tau pathology (Fig. S6g–j) in the mouse brains and found no differences between MSUT2 KO mice and wild-type littermates. In summary, our data show that MSUT2 regulates the seeding and/or spreading of different subtypes of neuronal tau pathology, while it has little effect on glial forms of tau pathology, further emphasizing the neuronal role of MSUT2 on tau pathogenesis.

Human neurodegenerative diseases usually present with multiple co-pathologies, and besides the defining tau and amyloid β (Aβ) pathologies, α-synuclein (α-syn) and TDP-43 inclusions are the most common types of co-pathologies in AD. To test if MSUT2 can regulate other forms of proteinopathy besides tau pathology, we crossbred MSUT2 KO mice with 5xFAD mice, which develop human-like Aβ plaques due to overexpression of mutant APP and PS1 genes [61]. Aβ plaque load was quantified in the 5xFAD/MSUT2 KO (5xKO) mice and 5xFAD mice with normal MSUT2 expression mice (5xWT) by quantifying the area occupancy of Aβ pathology and the number of plaques in the mouse brains (Fig. 3g). No significant difference was found in either measure between the 5xKO and 5xWT mice at 8 months of age (Fig. 3h, i). To test if MSUT2 is involved in α-syn-related pathways, we injected mouse α-synuclein (α-syn) preformed fibrils (mSyn-pffs) into the MSUT2 KO mice and wild-type littermates and evaluated α-syn pathology immunohistochemically using an anti-phospho-α-syn (EP1536Y) antibody at 1 m.p.i. Again, we found no significant changes in α-syn pathology in MSUT2 KO mice compared to wild-type littermates (Fig. 3j, k, Fig. S6 k, l). Taken together, these data support the idea that MSUT2 is specifically involved in tau pathogenesis and not in other forms of neurodegenerative proteinopathies.

Although MSUT2 has been previously shown to reduce tau pathology in transgenic mice, and here in human tau seeding mouse models, it is not known whether MSUT2 KO would still suppress tau pathology in the presence of the concurrent Aβ plaques found in AD brain, particularly the neuritic plaque (NP) tau pathology found in the vicinity of Aβ deposits. To investigate this, we injected AD-tau into the 5xKO and 5xWT mouse brains and quantified the amount of tau pathology at 1 m.p.i. Consistent with previous studies, AD-tau was found to induce NP tau pathology instead of NFTs in mice at this time point [33, 85] (Fig. 3l). Interestingly, we found that NP tau was significantly reduced in the AD-tau-injected 5xKO mice compared to the injected 5xWT mice (Fig. 3m), suggesting MSUT2-dependent tau seeding and/or spreading is observed both in the absence and presence of Aβ pathogenesis, with MSUT2 regulating NP, NFT and NT tau pathology.

As aging increases the risk of sporadic tauopathies, we examined whether aging could modulate the effect of MSUT2 KO on tau pathogenesis. MSUT2 KO mice and age-matched wild-type littermates were injected with AD-tau at 12—15 months of age, and the amount of tau pathology was analyzed at 3 m.p.i. (Fig. 3n). Our results reveal that the number of NFTs (Fig. 3o) was significantly reduced in the aged MSUT2 KO mice, and the total amount of tau pathology (NFTs + neuritic tau pathology) showed a non-significant trend toward reduction in aged MSUT2 KO mice (Fig. 3p). Altogether, our data suggest that MSUT2 regulates pathways in tau seeding and/or spreading independent of other proteinopathies, and that the effect of MSUT2 on tau pathology is still observed with advanced age, although it may be somewhat reduced.

### MSUT2 regulates pathogenic tau internalization in neurons

**Fig. 4:**
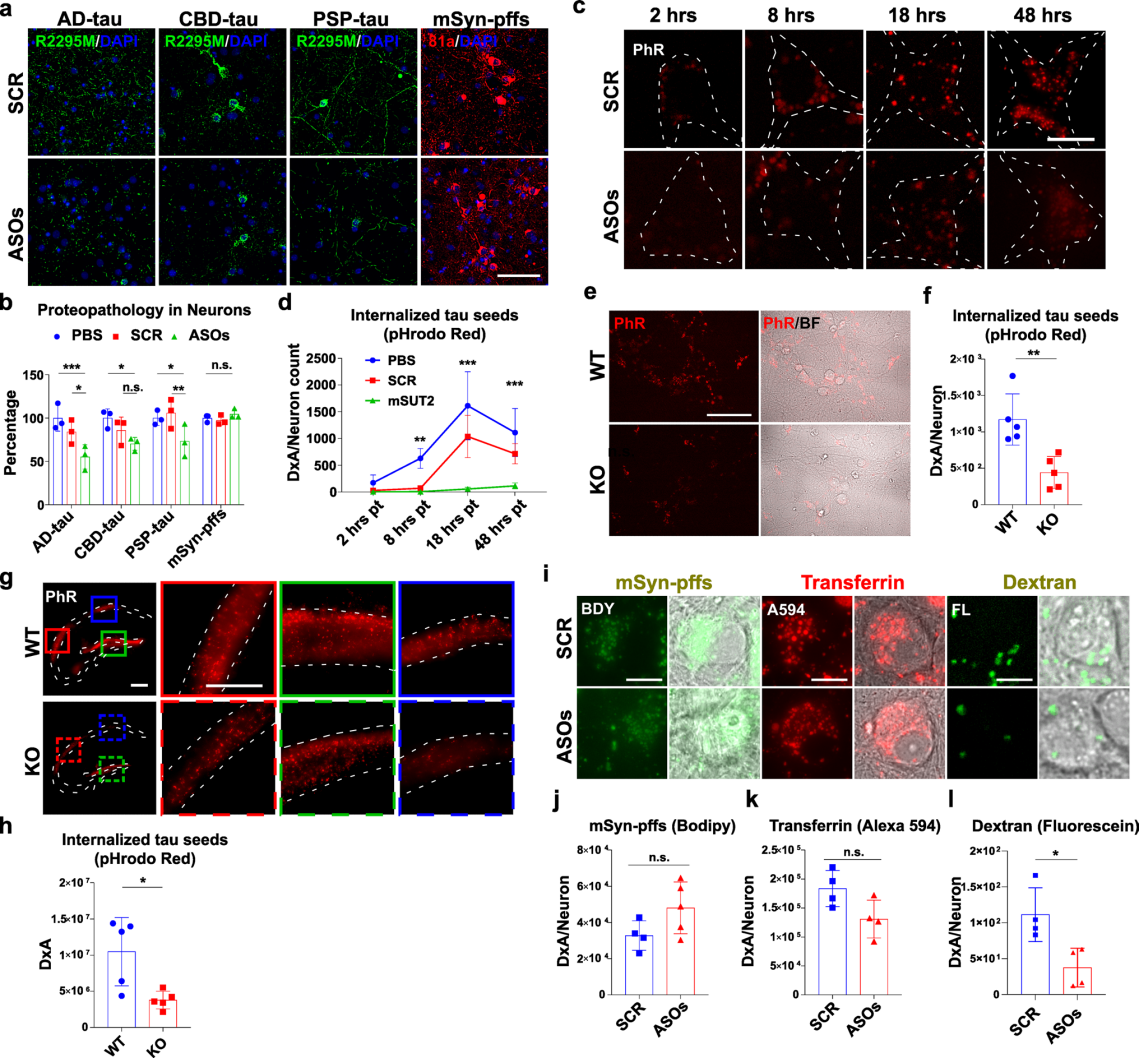
MSUT2 modulates the internalization of pathogenic tau seeds in neurons. **a** Representative images showing wild-type mouse primary neurons that were immunocytochemically stained with R2295M antibody (mouse tau, green) and DAPI (blue) to assess the amount of mouse tau pathology induced by human tau seeds. Wild-type mouse primary neurons were pretreated with antisense oligonucleotides (ASOs) against MSUT2 or scrambled control ASO (SCR) at DIV2 and human-derived tau seeds or mSyn-pffs were added at DIV7. Cells were extracted with detergent to remove soluble proteins and fixed at DIV21. Scale bar = 250 µm. **b** Quantification of R2295M immunoreactivity of each condition represented in **a**. Data were quantified using the fluorescent density x area of occupancy/DAPI count and normalized to the PBS-treated samples (as 100%). *P < 0.05, **P < 0.01, ***P < 0.001, n.s., not significant by two-way ANOVA followed by Tukey’s multiple comparisons test, *n* = 3 biologic repeats per group. Data were normalized to PBS-treated neurons in each group. Error bars represent the standard deviation. **c** Representative images showing neurons treated with MSUT2 ASOs (ASOs) or scrambled control ASO (SCR) at DIV2 and treated with pHrodo red dye (PhR)-labeled amplified AD-tau seeds (ADT40P1) at DIV7. PhR dye was detected at 2, 8, 18, and 48 h after the addition of ADT40P1. Scale bar = 5 µm. **d** Quantification of pHrodo red signal intensities from neurons treated as in **c**. Data are present as density x area/neuron count (DxA/Neuron count). The dashed line delineates the region corresponding to the neuron’s outline, as indicated by the brightfield channel. **P < 0.01, ***P < 0.001 by two-way ANOVA followed by Tukey’s multiple comparisons test, *n* = 3 biologic repeats per group. Error bars represent the standard deviation. **e** Representative images show MSUT2 KO and wild-type mouse primary neurons treated with pHrodo red (PhR)-labeled ADT40P1 at DIV7 and live imaged at 24 h post-treatment time. Scale bar = 25 µm. **f** Quantification of pHrodo red signal in neurons treated as in **e**. Data are present as density x area coverage/neuron count and normalized to WT neurons. **P < 0.01 by *t*-test, wild-type (WT) vs. MSUT2 KO (KO), *n* = 5 biologic repeats per group. Error bars represent the standard deviation. **g** Representative images show the uptake of fluorescently labeled tau seeds in MSUT2 KO and wild-type mouse brains in vivo. Mice were stereotactically-injected with pHrodo red-labeled ADT40P1 at 3 months of age. Mouse brains were quickly dissected at 2 d.p.i., sectioned, and imaged using live imaging microscopy. Representative images show the dorsal hippocampal regions of injected mice. Scale bar = 500 µm (overview) and 100 µm (insets). **h** Quantification of pHrodo red signal density x area of occupancy (DxA) in the hippocampal regions of the ADT40P1-injected MSUT2 KO and wild-type mice in sections stained as in **g**. **P < 0.01 by t-test, wild-type (WT) vs. MSUT2 KO (KO), *n* = 5 mice per group. Error bars represent the standard deviation. **i** Representative images showing wild-type primary neurons that were treated with MSUT2 ASOs (ASOs) or scramble controls (SCR) at DIV2 and treated with bodipy (BDY)-labeled mSyn-pffs (mSyn-pffs), Alexa594 (A594)-labeled transferrin, or fluorescein (FL)-labeled dextran (500 kDa) at DIV7, with imaging 24 h later. Internalized proteins were revealed using live cell microscopy. Extracellular bodipy and fluorescein fluorescence were quenched by 500 mM trypan blue before imaging. Representative images show fluorescent signals for bodipy, Alexa594, and fluorescein as well as fluorescence images merged with bright field images. Scale bar = 5 µm. **j**–**l** Quantification of fluorescent signal intensities (DxA) in each condition in neurons stained as in **i**. n.s., not significant, *P < 0.05 by *t*-test, MSUT2 ASOs (ASOs) vs. scramble controls (SCR), *n* = 4–5 biologic repeats per group. Error bars represent the standard deviation

To determine the mechanism(s) of how MSUT2 modulates the seeding and spreading of tau pathology, we used antisense oligonucleotides (ASOs) against MSUT2 to knock down (KD) MSUT2 expression in primary mouse neurons. We first validated the effects of six different ASO sequences on MSUT2 protein expression (Fig. S7a) and found that all 6 ASOs can significantly suppress MSUT2 protein expression in wild-type mouse primary neurons (Fig. S7b). When we co-treated the neurons with MSUT2 ASOs and pathogenic protein seeds from AD, CBD, or PSP brains, or mSyn-pffs, we found that tau inclusions, but not α-syn aggregates, were reduced in the ASO-treated neurons compared to PBS- or scrambled sequence control (SCR)-treated neurons (Fig. 4a, b). We also confirmed that the amount of insoluble tau was reduced in the ASO-treated neurons using immunoblots (Fig. S8a, b). These data are consistent with the aforementioned in vivo observations in which loss of MSUT2 expression reduces tau pathology.

To further examine the effect of MSUT2 on tau seeding and spreading, we adapted a recently developed cell-free amplification method for amplifying AD-tau that allowed for the generation of fluorescent-labeled recombinant tau seeds (Fig. S9a). Neuron-based activity tests showed that AD-tau seeds that were amplified with recombinant T40 tau containing the pH-insensitive hylite488 fluorescent label (ADT40P1-h488) could induce mouse tau pathology like the parent AD-tau seeds (Fig. S9b). Using a previously described protocol [41], we specifically quenched the fluorescent signal coming from extracellular seeds (green) using trypan blue (Fig. S9c), which allows for the visualization of internalized intracellular fluorophore-tagged tau seeds during live imaging. The amount of internalized tau seeds in neurons was quantified based on the integrated intracellular fluorescence as a function of time after seeds addition (Fig. S9d, e), and we observed that the half-life of internalized ADT40P1-h488 was twice as long (111.6 h) as T40 monomers (51.5 h) (Fig. S9e). This indicates that the assembled fibrillar tau has significantly greater resistance to degradation than monomeric tau.

The internalization of ADT40P1-h488 tau seeds was examined in neurons treated with or without ASOs against MSUT2 (Fig. S10a). Intracellular fluorescent signal was significantly decreased in MSUT2 ASO-treated neurons compared to PBS or SCR-treated neurons after 24 h post-treatment time (Fig. S10b), suggesting a global reduction of internalized tau seeds in the MSUT2 KD neurons. Using an ADT40P1 preparation made with a pH-sensitive dye (pHrodo™ red; PhR), we used time-lapse live imaging microscopy to measure the progression of tau seeds into acidic compartments such as late endosomes and lysosomes, and internalized tau seeds could be observed in acidic compartments as early as 8 h post-treatment time. Notably, the amount of internalized tau seeds that progressed to acidic compartments was reduced in the MSUT2 ASOs-treated neurons compared to controls (PBS and SCR), in agreement with the global reduction of pathogenic tau seed uptake (Fig. 4c, d). These phenotypes could also be reproduced using MSUT2 KO mouse primary neurons (Fig. 4e, f) and importantly, in vivo after injection of ADT40P1-PhR into the adult MSUT2 KO mouse brains (Fig. 4g, h). Interestingly, in the latter in vivo studies, the majority of pathogenic tau seeds were taken up by cells located in the CA and dentate gyrus regions (Fig. 4g), confirming the central role of neurons in tau uptake. To reveal the endocytic pathways that MSUT2 regulates, we employed different macromolecules that enter neurons via differential endocytic routes in primary neurons (Fig. 4i), including mSyn-pffs (receptor-mediated endocytosis [11, 54] and macropinocytosis [4]), transferrin (clathrin-mediated endocytosis [77]), and dextran (100 kDa; macropinocytosis [50]) and measured their uptakes in the primary neurons treated with MSUT2 or control ASOs. We did not observe any significant change in the internalization of these other protein species (Fig. 4j, k) except for dextran (Fig. 4l), suggesting that MSUT2 specifically regulates tau internalization by modulating neuronal macropinocytosis.

### Gene expression analyses after neuronal MSUT2 KO identifies multiple genes implicated in tau pathogenesis

As an RNA binding protein, it is known that MSUT2 binds to the Poly(A) tails of mRNA with its CCCH finger domains and regulates mRNA stability [70]. To specifically identify the mRNAs under the influence of MSUT2 in neurons, we performed single-cell metabolically labeled new RNA tagging sequencing (scNT-seq) in primary neuron cultures of wild-type (WT) and MSUT2 KO mice. In addition to discriminating cell type-specific changes, the scNT-seq technique allows for a comparison of transcript synthesis vs. turnover in the presence or absence of MSUT2. Accordingly, primary neuron cultures from MSUT2 KO mice and wild-type littermates were metabolically labeled at DIV 10 and the transcriptome and differentially expressed genes (DEGs), as well as mRNA stabilities (represented as synthesis/degradation ratio), were elucidated in the different cell culture populations.

**Fig. 5:**
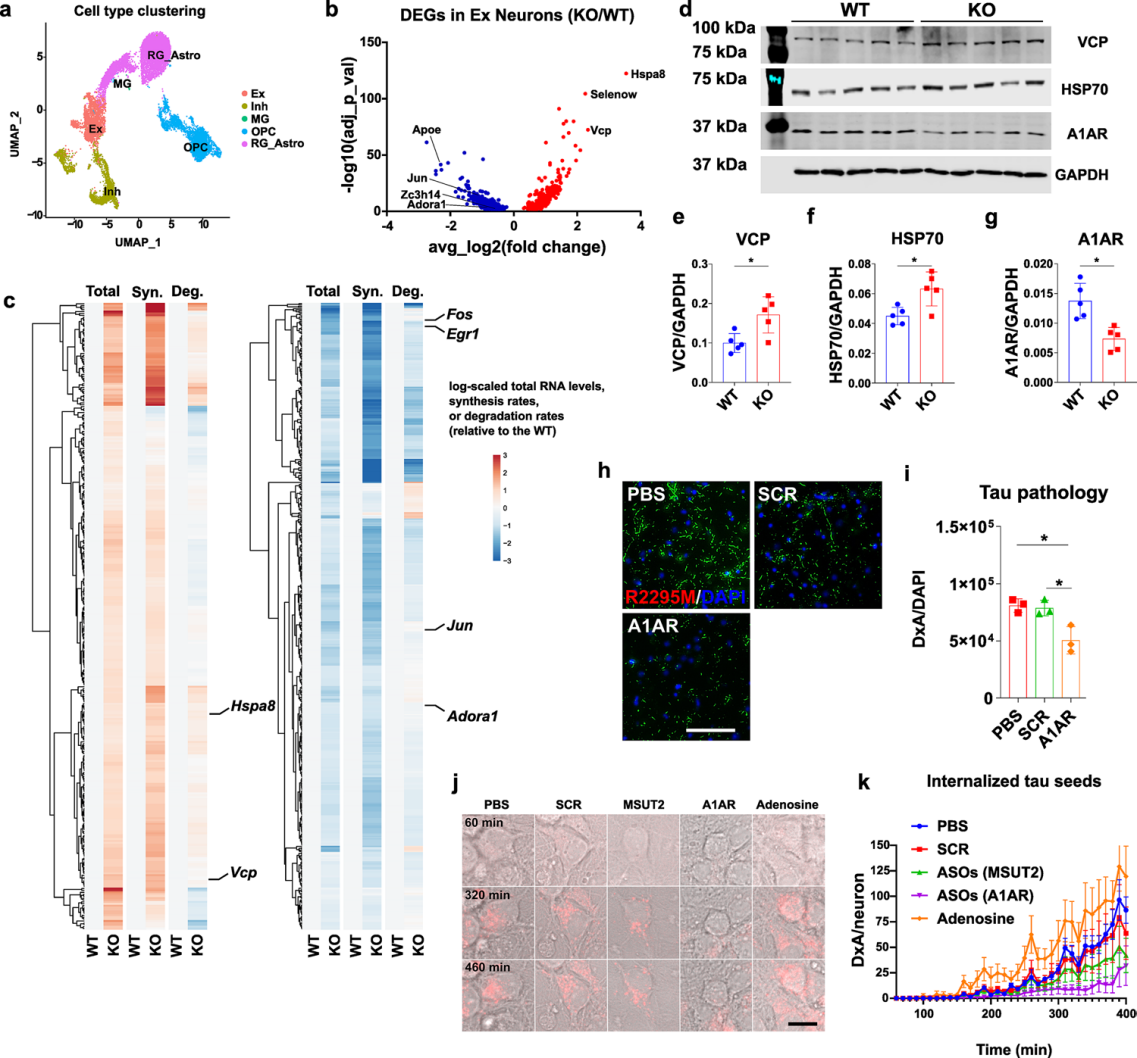
MSUT2 modulates tau pathogenesis via adenosine signaling. **a** UMAP plot shows distinct cell populations in scNT-sequencing of wild-type and MSUT2 KO primary neuron cultures at DIV 10. Cell populations were determined by expression levels of multiple genes in each cell type. Ex = excitatory neurons, Inh = inhibitory neurons, MG = microglia, OPC = oligodendrocyte precursor cells, RG_Astro = radial glia astrocytes. **b** Volcano plot showing differentially expressed genes (DEGs) in wild-type (WT) and MSUT2 KO (KO) excitatory neurons identified by scNT-seq. WT and KO primary neurons were cultured and collected at DIV10 in single-cell resuspension and sequenced using scNT-seq. DEGs are shown as WT vs. KO neurons. DEGs with adjusted *p* < 0.01 are shown on the graph. Increased DEGs (KO greater than WT) are in red while decreased DEGs are in blue. **c** Heatmaps showing the total RNA level, synthesis rate (syn) and degradation rate (deg) changes of DEGs in excitatory neurons with (WT) or without (KO) expression of MSUT2. Values of DEGs in WT neurons were set as 0. The color hue indicates the level of changes, with red indicating increases (greater in KO than WT) and blue indicating decreases. **d** Immunoblots show VCP, HSP70 and A1AR protein expression levels in MSUT2 KO (KO) and wild-type (WT) littermates at 3 months of age. GAPDH was used as the loading control. *n* = 5 mice per group. **e**–**g** Quantification of VCP, HSP70, A1AR optical density from immunoblots as depicted in **d**. Data were normalized to the GAPDH signal. *P < 0.05 by t-test, MSUT2 KO (KO) vs. wild-type (WT) mice, *n* = 5 mice per group. Error bars represent the standard deviation. **h** Representative images showing mouse tau pathology induced by AD-tau in neurons treated with PBS, ASOs for A1AR, or scrambled control ASO. Primary neurons were transfected with ASOs at DIV2 and treated with AD-tau at DIV7. Insoluble mouse tau aggregates were revealed with R2295M antibody. DAPI reveals nuclei. Scale bar = 50 µm. **i** Quantification of mouse tau pathology (area x density/DAPI count) in primary neurons as depicted in **h**. *P < 0.05, **P < 0.01, ***P < 0.001 by one-way ANOVA followed by Tukey’s multiple comparison, *n* = 4 biologic repeats/condition. Error bars represent the standard deviation. **j** Representative time-lapse live images of internalized tau seeds in primary neurons treated with PBS, scrambled control ASOs (SCR), MSUT2 ASOs (MSUT2), A1AR ASOs (A1AR) at DIV2, or adenosine at DIV6, with pHrodo red-labeled ADT40P1 added at DIV7. Time in the figures is the interval after the addition of labeled ADT40P1. Scale bar = 5 µm. **k** Quantification of internalized pHrodo red signal (area x density/cell count) as a function of time, as depicted in **j**. *n* = 4 biologic repeats/condition. Error bars show standard errors

After filtering low-quality cells, we retained 4855 and 2624 cells from wild-type and MSUT2 KO samples, respectively. We identified five major cell types, including excitatory neurons (Ex), inhibitory neurons (Inh), and non-neuronal cell types based on their characteristic gene expression (Fig. 5a). Loss of MSUT2 differentially affects the transcriptome in different cell populations, indicating a cell type-specific role of MSUT2 (Fig. S11a). In neurons, these genes were predicted to be involved in multiple pathways, including those associated with neurodegenerative disease (Fig. S11b). Interestingly, in line with our tau internalization data, genes controlling endocytosis were also found to be altered in the MSUT2 KO neurons (Fig. S11b). Notably, several previously reported genes protective against misfolded proteins or oxidative stresses, such as *Hspa8* [46, 87], *Selenow* [87], and *Vcp* [16] are the most significantly up-regulated across all the MSUT2 KO cell populations in the culture, whereas genes such as *Adora1* [18, 40, 63]*, Apoe* [74] and *Jun* [37] that have been implicated in tau toxicity are specifically down-regulated in MSUT2 KO excitatory neurons (Fig. 5b, Data 2). These results suggest that MSUT2 regulates multiple transcripts that may play a direct or indirect role in tau pathogenesis in a cell type-dependent manner. When we compared the DEGs in excitatory neurons from MSUT2 KO cultures with a recent single-cell sequencing study of AD patient excitatory neurons [55], we found 61 overlapping genes, suggesting that there are a number of gene transcripts that show similar dysregulation in AD and after MSUT2 KO (Fig. S11c). By taking advantage of scNT-seq, we estimated the mRNA synthesis and degradation rate in different cell types and calculated the mRNA synthesis and degradation rates of 329 up-regulated genes and 494 down-regulated genes in excitatory neurons (Fig. 5c, Data 3). In general, 97.0% (319 out of 329) of the up-regulated DEGs show increased synthesis rates (synthesis: KO/WT > 1) while only 1.2% (6 of the 494) of the down-regulated DEGs show increased synthesis rates. The fact that these genes are down-regulated indicates that there is an enhancement of their turnover that exceeds the increase in synthesis rate. There were 37.3% of the up-regulated DEGs vs. 18.2% of down-regulated DEGs that show increased degradation rates in the KO neurons. The overall transcript level is ultimately influenced by the balance of synthesis and degradation rates, and these findings suggest that alterations in synthesis rates predominantly drive the regulation of DEGs in the MSUT2 KO neurons. In summary, our data strongly imply that MSUT2 modulates the stability of numerous genes across distinct cell populations, and some of these genes might play a role in tau pathogenesis and perhaps tau endocytosis.

### MSUT2 regulates tau endocytosis via adenosinergic signaling

As noted, a number of MSUT2-regulated neuronal genes were identified that have previously been implicated in the regulation of tau pathology, including *ApoE*, *VCP*, *Selenow* and *Hspa8*. While the changes in expression of one or more of these gene products might contribute to the observed reduction of tau pathology observed after MSUT2 KO, it is not clear based on the known functions of these proteins that they play a role in the observed reduction of macropinocytosis and tau seed uptake observed after MSUT2 KD or KO. In this regard, the downregulation of *Adora1* (encoding adenosine receptor 1) after MSUT2 KO is noteworthy, as the literature suggests a potential linkage of Adora1 with tauopathy, as well as an involvement in neuronal endocytosis [18, 40, 63, 66]. To validate the transcriptomic changes, we biochemically measured the protein expression levels of the DEGs VCP (*Vcp*), HSP70 (*Hspa8*) and A1AR (*Adora1*) in adult 3-month-old MSUT2 KO mouse brains (Fig. 5d–g). The immunoblots confirmed that these proteins are consistently changing in the same way (increased or decreased) in the MSUT2 KO mouse brain as the transcripts changed in MSUT2 KO neuron cultures. To further investigate the contribution of gene expression in tau spreading, we correlated the brain regional changes of tau pathology between WT and MSUT2 KO mice with the spatial expression of DEGs in MSUT2 KO neurons from a previously published dataset [48] and identified A1AR expression as being highly correlated with tau spreading (Data 4). This finding led us to explore the mechanisms by which A1AR regulates tau endocytosis.

To examine if A1AR might regulate tau pathology, we first treated primary neurons with an adenosine receptor agonist (adenosine) or antagonist (caffeine), followed by treating with AD-tau seeds and subsequent measurement of induced mouse tau aggregates using a mouse tau-specific antibody (R2295M) (Fig. S12a). We observed clear dose-dependent changes in tau pathology after treatment with these pharmaceutical compounds. Specifically, the adenosine receptor agonist, adenosine, increased the amount of tau pathology (Fig. S12b) while the antagonist, caffeine, decreased pathology (Fig. S12c). These results suggest that adenosine receptor binding can influence the AD-tau-seeded tau pathology in neurons. Moreover, when the neurons were co-treated with both agonist and antagonist, their effects were neutralized (Fig. S12d), confirming the modulatory effect was unlikely due to an off-target effect of the compounds. When we treated the neurons with these compounds and measured the internalization of fluorescently labeled pathogenic tau seeds (ADT40P1-PhR), we found that caffeine treatment significantly decreased the amount of internalized tau seeds while adenosine increased it (Fig. S12e and f), which correlates with the changes in AD-tau-seeded tau pathology in the primary neuron model. Notably, the same treatment was less effective in the MSUT2 KO mouse primary neurons, suggesting that an adenosine receptor, likely A1AR, is the downstream effector needed for the internalization of tau seeds (Fig. S12g and h).

As the pharmacologic agents used above act on both A1AR and A2AR receptors, we wanted to further confirm the role of A1AR in tau seeding. Utilizing ASOs to A1AR, we found that A1AR KD led to a decrease in AD-tau-seeded pathology relative to neurons treated with scrambled control ASOs (Fig. 5h and i). Similar to neurons treated with ASOs against MSUT2, neurons treated with ASOs against A1AR showed reduced internalization of tau seeds (Fig. 5j and k). Notably, modulating A1AR activity or MSUT2 level did not significantly alter the bulk mitochondria or lysosome densities or morphologies in these neurons (Fig. S13), suggesting the MSUT2-A1AR pathway is unlikely affect tau seeding via changing mitochondrial and lysosomal activities. These data indicate that A1AR is a downstream target of MSUT2 that modulates endocytosis of pathogenic tau seeds.

### A1AR regulates tau endocytosis via ASAP1 signaling

**Fig. 6:**
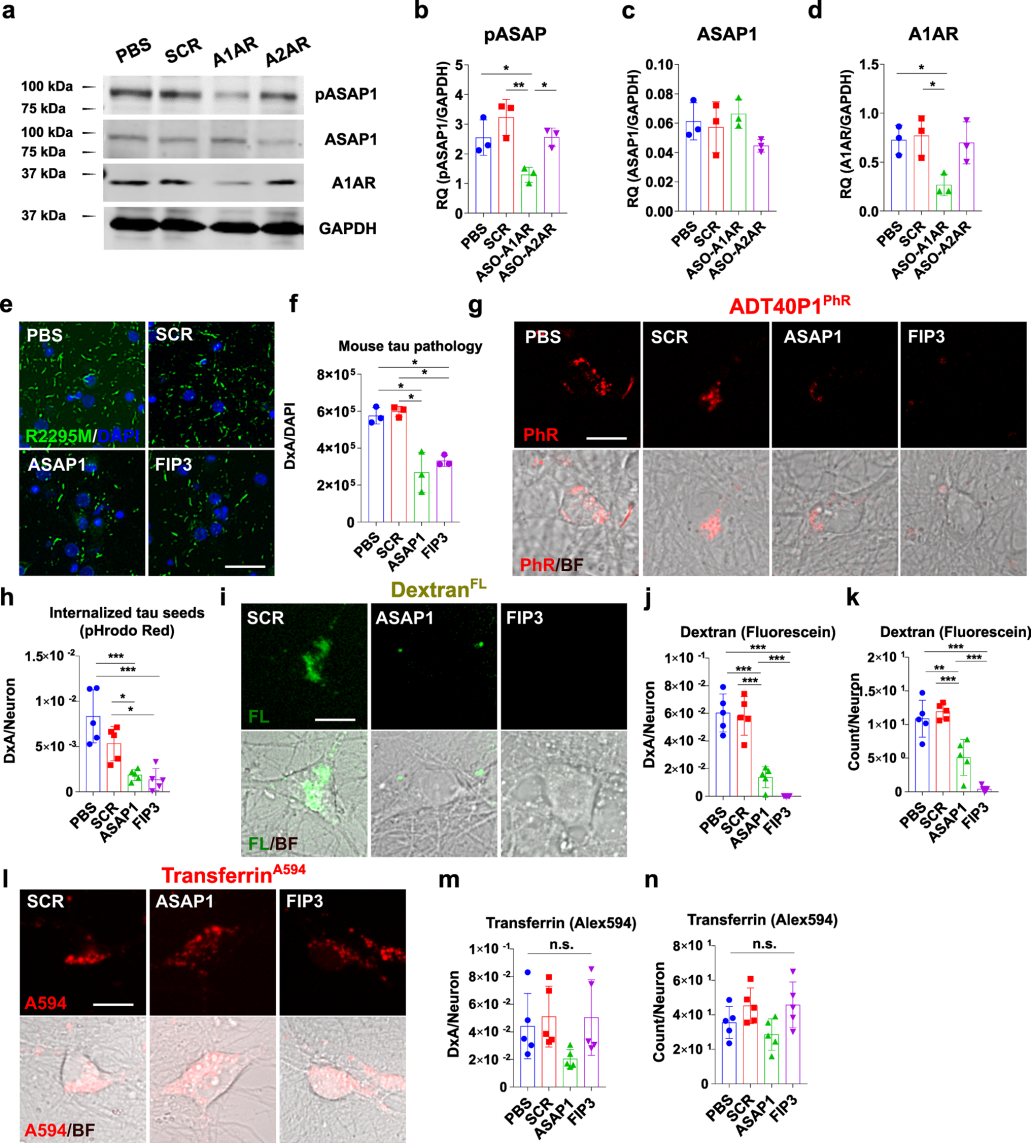
Adenosine signaling regulates ASAP1 and micropinocytosis. **a** Mouse primary neurons were treated with ASOs against A1AR or A2AR, scrambled ASO control (SCR) or PBS at DIV2. Cells were harvested at DIV7, and cell lysates were probed with pASAP1, ASAP1, A1AR and GAPDH antibodies by immunoblots. **b**–**d** Quantification of pASAP1, ASAP1, A1AR, GAPDH immunoreactivity as shown in **a**. *P < 0.05, **P < 0.01 by one-way ANOVA followed by Tukey’s post hoc test, *n* = 3 biologic repeats. Error bars represent the standard deviation. **e** Representative images showing mouse tau pathology induced by AD-tau in neurons treated with PBS, ASAP1 ASOs (ASAP1), FIP3 ASOs (FIP3), or scrambled control ASO (SCR). Primary neurons were transfected with ASOs at DIV2 and treated with AD-tau at DIV7. Insoluble mouse tau aggregates were revealed with R2295M antibody. DAPI reveals nuclei. Scale bar = 50 µm. **f** Quantification of mouse tau pathology (area x density/DAPI count) in primary neurons as depicted in **e**. *P < 0.05 by one-way ANOVA followed by Tukey’s multiple comparison, biologic repeats/condition. Error bars represent the standard deviation. **g** Representative live images of internalized tau seeds in primary neurons treated with PBS, scrambled control ASO (SCR), ASAP1 ASOs (ASAP1), or FIP3 ASOs (FIP3) at DIV2, with pHrodo red-labeled ADT40P1 (ADT40P1-PhR) added at DIV7. Live cell images were taken at DIV8 (24 h post-treatment). Scale bar = 5 µm. **h** Quantification of internalized tau seeds as measured by pHrodo red signal, as depicted in **g**. *P < 0.05, **P < 0.01, ***P < 0.001 by one-way ANOVA followed by Tukey’s multiple comparison, n = 5 biological repeats/condition. Fluorescent density x Area was normalized to Neuron count (DxA/Neuron). Error bars show standard errors. **i** Representative live images of internalized tau seeds in primary neurons treated with scrambled control ASO (SCR), ASAP1 ASOs (ASAP1), or FIP3 ASOs (FIP3) at DIV2, with fluorescein-labeled Dextran added at DIV7. Live cell images were taken at DIV8 (24 h post-treatment). Extracellular signals were quenched using 100 μM trypan blue before imaging. Scale bar = 5 µm. **j**, **k** Quantification of internalized Dextran as measured by fluorescein signal, as depicted in **i**. *P < 0.05, **P < 0.01, ***P < 0.001 by one-way ANOVA followed by Tukey’s multiple comparison, *n* = 5 biological repeats/condition. Fluorescent density x area or counts of endocytosed puncta (count) were normalized to Neuron count (DxA/Neuron or count/Neuron). Error bars show standard errors. **l** Representative live images of internalized tau seeds in primary neurons treated with scrambled control ASO (SCR), ASAP1 ASOs (ASAP1), or FIP3ASOs (FIP3) at DIV2, with Alexa594-labeled Transferrin added at DIV7. Live cell images were taken at DIV8 (24 h post-treatment). Scale bar = 5 µm. **m**, **n** Quantification of internalized transferrin as measured by Alex594 signal, as depicted in **l**. *n* = 5 biological repeats/condition. n.s. by one-way ANOVA followed by Tukey's multiple comparison, *n* = 5 biological repeats/condition. Fluorescent density x area or counts of endocytosed puncta (count) were normalized to Neuron count (DxA/Neuron or count/Neuron). Error bars show standard errors

Since macropinocytosis appears to be the major pathway involved in tau endocytosis in our models, we searched for potential effectors under the control of A1AR that may contribute to macropinocytosis. Among the candidates, we noticed ASAP1 as a potential regulator of endocytosis, specifically macropinocytosis, as reported in previous studies [7, 51]. Notably, ASAP1 was also identified as a potential genetic risk factor for PSP [9, 73, 86], a form of primary tauopathy. Therefore, our first step was to test the activation and expression of ASAP1 in A1AR KD mouse primary neurons. The results showed that downregulation of A1AR decreased the activated form of ASAP1 (pY782) in the neurons, while the total amount of ASAP1 was not affected (Fig. 6a–d). To confirm the relationship between MSUT2, A1AR, and activated pASAP1 in the MSUT2 KO mice, we measured neuronal levels of these proteins in MSUT2 KO and wild type mouse brain by immunofluorescence staining and found that A1AR and pASAP1 are down-regulated in the MSUT2 KO mouse brains compared to their wild type littermates (Fig. S14a and b). Moreover, their expression levels positively correlate with each other in the wild-type mouse brains (Fig. S14c–e). These results further validated the regulatory role of MSUT2 on A1AR and pASAP1). Similar to the immunoblots (Fig. S2a, b), there is non-functional MSUT2 protein expression in the MSUT2 KO mouse brains. Previous reports indicated that ASAP1 regulates macropinocytosis through FIP3 and RAB11 [36]. Building upon these findings, we investigated tau seeding in neurons after knockdown of FIP3 and ASAP1 in wild-type mouse primary neurons. Notably, tau pathology induced by AD-tau seeding was significantly reduced in neurons treated with ASAP1 or FIP3 ASOs compared to those treated with PBS or scrambled control ASOs (Fig. 6e, f), suggesting the involvement of ASAP1 in tau seeding. We further assessed tau internalization in neurons after downregulation of ASAP1 and FIP3, and a reduction of tau internalization was observed in neurons treated with ASAP1 and FIP3 ASOs (Fig. 6g, h). Consistent with our observation in MSUT2 KD neurons (Fig. 4k, l), both ASAP1 and FIP3 appear to regulate macropinocytosis as revealed by the effects on internalization of dextran (Fig. 6 i–k). There is also a non-significant trend toward reduction of transferrin uptake after ASAP1 KD, as there was for MSUT2 KD (Fig. 4k), although FIP3 KD did not affect clathrin-mediated transferrin internalization (Fig. 6m, n). Thus, although the reduction of MSUT2 and ASAP1 might affect clathrin-mediated endocytosis in addition to macropinocytosis, the fact that FIP3 KD reduces tau internalization without affecting transferrin uptake further suggests that the MSUT2, ASAP1 and FIP3 effects on tau internalization are via macropinocytosis. In summary, our data suggests that A1AR is a downstream MSUT2-regulated gene product that, through regulation of ASAP1 activation, controls the internalization of tau seeds.

## Discussion

Previous studies have indicated that MSUT2 KO does not substantially impact the lifespan and brain function of mice. However, it does lead to a reduction in tau pathology and the amelioration of brain dysfunction in transgenic *C. elegans* and mice that overexpress mutant human tau [30, 82, 83]. This suggests a potential involvement of MSUT2 in regulating tau pathogenesis and associated neuronal dysfunction in mouse models of tauopathy. In line with this observation, our research reveals a correlation between neuronal MSUT2 expression levels and the extent of tauopathy in human patients. Moreover, KO of MSUT2 can inhibit the spatiotemporal spreading of tau pathology in mice that was induced by patient-derived tau seeds in a connectome-dependent manner, further establishing a neuronal role of MSUT2 on tau pathogenesis. The MSUT2 KO effect on tau pathology was also observed in mouse models seeded with different tau strains and was independent of Aβ plaque co-pathology. In fact, loss of MSUT2 did not influence Aβ plaque or α-syn pathology. The inhibitory effect of MSUT2 reduction on tau pathology could also be reproduced in primary neuron cultures using either ASOs to MSUT2 or in MSUT2 KO primary neurons. Using live cell imaging and fluorescently labeled tau seeds, we showed that downregulation of MSUT2 significantly reduced the internalization of pathogenic tau seeds in neurons in vitro and in vivo. To elucidate the molecular mechanisms of this MSUT2 effect, we identified DEGs under the control of MSUT2 in neurons and validated a downstream target gene, adora1, as a regulator of macropinocytosis and pathogenic–tau internalization that acts through activation of ASAP1. We have thus revealed a link between A1AR and ASAP1 in macropinocytosis and uncovered a novel mechanism of tau uptake regulation associated with ASAP1, a potential genetic risk factor for PSP. Importantly, these findings indicate that A1AR and ASAP1 could serve as potential therapeutic targets for the treatment of tauopathies.

Although a previous study [83] revealed that MSUT2 KO could reduce tau pathology in a transgenic mouse tauopathy model, the mechanism by which MSUT2 reduction led to lower tau pathology was unclear. Our utilization of seeded models of tau pathologic spreading allowed for further dissection of the role of MSUT2 in tau pathogenesis. The use of tau spreading models provides several advantages compared to transgenic models: (1) the development of tau pathology does not depend on tau overexpression; (2) the onset of tau pathology can be spatiotemporally traced; and (3) The model is compatible with other transgenic or non-transgenic models, facilitating the study of multiple factors and co-pathologies on tau pathogenesis. Utilizing such models, we showed that loss of MSUT2 results in reduced spreading of tau pathology induced by different tau strains, with this suppressive effect observed up to 12 months after seeding with pathologic tau. Interestingly, the results from bigenic 5xFAD/MSUT2 KO mice injected with AD-tau showed that loss of MSUT2 can suppress neuritic plaque tau, indicating that MSUT2 can modulate all forms of seeded neuronal tau pathology. Notably, MSUT2 KO mice injected with PSP- and CBD-tau also showed a decrease in neuronal tau pathology, but not astrocytic tau pathology, suggesting that reduction of MSUT2 does not affect glial tau seeding and spreading. In addition, MSUT2 KO did not result in significant changes in markers of glial reactivity, suggesting that the effect of MSUT2 KO on neuronal tau pathology was likely not the result of overt changes in glial phenotype.

Our in vivo data indicate that loss of MSUT2 reduces the internalization of tau seeds, and multiple endocytic pathways have been suggested to be involved in tau internalization, including micropinocytosis [22, 35, 84], clathrin-mediated endocytosis [21], and tunneling nanotubes [75]. In addition, macropinocytosis has been suggested to comprise one major endocytic pathway for the internalization of oligomeric and fibrillar tau species [22, 35, 84]. These studies have identified multiple molecular species that may directly interact with tau protein to facilitate uptake, including heparan sulfate proteoglycans (HSPGs) [35], cellular prion protein (PrPC) [17], and low-density lipoprotein (LDL) receptor related protein 1 (LRP1) [14, 69]. Other studies suggest tau internalization may be via endocytic pathways that require LDL [69] and Bridging Integrator 1 (BIN1) [8]. Many of these prior studies of tau internalization utilized non-neuronal cellular systems and synthetic tau fibrils, and it is unclear if all these findings translate to neuron-mediated uptake of tau fibrils from diseased brain. In this regard, we chose to use primary neurons and human-like pathogenic tau seeds in the present study in an attempt to provide a disease-relevant model system. This included modification of our previous human pathologic tau amplification protocol [85] to generate fluorescently labeled tau seeds that recapitulate the pathogenicity of human tau seeds. To our knowledge, these are the first fluorescently labeled tau seeds that mimic the pathogenicity of human tau. Using these tools, we found that the internalization of tau seeds was decreased in MSUT2 knockout neurons. This reduction of tau seed uptake in neurons correlated with the decline of tau pathology formation in the neuron cultures, and a similar lowering of fluorescently labeled tau seed internalization was also reproduced in vivo in MSUT2 KO mice. Interestingly, we did not observe a similar effect of MSUT2 KO on α-syn pffs uptake in neurons. This could be due to the use of non-human-derived pffs and/or that internalization of α-syn pffs and tau seeds are mediated by different endocytosis pathways. Future testing with human-derived α-syn seeds may provide further insights into α-syn uptake mechanism(s).

To identify the molecular mechanism by which MSUT2 regulates tau internalization, we leveraged a newly developed single nuclear sequencing technique [67], which allows us to label newly synthesized mRNAs and measure the synthesis and turnover rate of the target mRNAs in neurons. The approach led to the discovery of genes with altered expression in MSUT2 KO neurons, demonstrating a modulatory role of MSUT2 on specific mRNAs. Among the DEGs regulated by MSUT2 were those encoding tau kinases and chaperone proteins associated with the protein misfolding machinery. This observation suggests that MSUT2 might oversee various cellular processes that could influence tau pathogenesis, and gaining an understanding of the relationship of these DEGs to tau pathology would require further investigation. However, here we focused on one of the down-regulated DEGs in MSUT2 knockout excitatory neurons, adora1, that has been implicated in AD and the regulation of endocytosis. Adora1 encodes the adenosine receptor 1 (A1AR), which belongs to a family of cell membrane [63] receptors that govern adenosinergic signaling pathways. Notably, A1AR levels were previously reported to be associated with AD and dementia [1, 40], and the consumption of the A1AR antagonists caffeine and theophylline is negatively associated with the onset of AD and dementia [10, 25]. Interestingly, A1AR antagonists also reduce phosphorylated tau levels [47] and rescue synaptic dysfunction [18] in transgenic mouse models. Moreover, adenosine signaling was reported to mediate the endocytosis of AMPA receptors [66], suggesting a potential role in endocytic pathways. The influence of A1AR on tau pathogenesis was examined using known adenosine receptor agonists and antagonists and the results revealed that inhibition of adenosine receptor signaling can effectively suppress AD-tau-seeded neuronal tau pathology and reduce the internalization of pathogenic tau seeds, similar to the downregulation of MSUT2. Importantly, these results were reproduced with ASOs directed to A1AR but not to A2AR, indicating that the effect of the tested adenosine receptor ligands was via A1AR binding. Since A1AR has not been identified as a tau interactor [78] and we observed a similar effect of A1AR antagonism on the internalization of dextran, we propose that A1AR facilitates tau internalization via macropinocytosis. As one of the potential genetic risk factors for PSP, the mechanism of ASAP1 involvement in tau pathology has not been previously investigated. We found ASAP1 is a downstream effector of A1AR that regulates the endocytosis of tau seeds and tau seeding in recipient neurons. Previous studies have indicated an overlap of binding ligands and downstream effectors of A1AR and A3R (adenosine A3 receptor), both of which belong to the Gi family of receptors [5, 6, 52]. There is limited expression of A3R in mouse primary neurons [23, 52], so our study cannot definitively rule out concurrent involvement of both A1AR and A3R on the modulation of ASAP1 in the adult mouse brain. Nevertheless, we did observe a differential effect of A1AR and A2AR in our experiments, which aligns with their distinct roles in the central nervous system.

In summary, we show that decreased MSUT2 expression reduces the transmission of tau pathology in tau spreading models by limiting the internalization of pathogenic tau seeds through a mechanism that depends, at least in part, on reduced A1AR function (see schematic model in Fig. S15). MSUT2 regulates the expression of A1AR, the downregulation of which exhibits a similar modulatory effect on pathogenic tau internalization as reduced MSUT2 expression in mouse neurons. Our study thus provides novel insights into the role of MSUT2 in tau pathogenesis, including the involvement of the A1AR and ASAP1 signaling pathway. This suggests A1AR or ASAP1 may be promising therapeutic targets to reduce the progression of tau pathology in human tauopathies.

### Supplementary Information

Below is the link to the electronic supplementary material.

Supplementary file1 (DOCX 27,466 KB)

Supplementary file2 (XLSX 520 KB)

## Data Availability

All the supporting data are included in the electronic supplementary material. Experimental models and materials in the study can be shared via proper material transfer agreements for research purposes.
