# Supplementary material for: MSUT2 regulates tau spreading via adenosinergic signaling mediated ASAP1 pathway in neurons
Source: Acta Neuropathol. 2024 Mar 12;147(1):55. doi: 10.1007/s00401-024-02703-3 (PMC10933148; doi:10.1007/s00401-024-02703-3)
Supplement: Supplementary file 1 — Supplementary file1 (DOCX 27,466 KB) [file 401_2024_2703_MOESM1_ESM.docx]

**Supplementary Information**

***MSUT2 Regulates Tau Spreading via Adenosinergic Signaling Mediated ASAP1 Pathway in Neurons***

Hong Xu^1,^ *, Qi Qiu^2^, Peng Hu^3^, Kevt’her Hoxha^1^, Elliot Jang^1^, Mia O’Reilly^1^, Christopher Kim^1^, Zhuohao He^4,5^, Nicholas Marotta^1^, Lakshmi Changolkar^1^, Bin Zhang^1^, Hao Wu^2^, Gerard D. Schellenberg^6^, Brian Kraemer^7,8,9^, Kelvin C. Luk^1^, Edward B. Lee^10^, John Q. Trojanowski^1,^ **^†^** , Kurt R. Brunden^1^, Virginia M.-Y. Lee^1,^ *

**From**

^1^Department of Pathology and Laboratory Medicine, Institute on Aging and Center for Neurodegenerative Disease Research, Perelman School of Medicine at the University of Pennsylvania, PA, USA.

^2^Department of Genetics, Penn Epigenetics Institute, Institute of Regenerative Medicine, University of Pennsylvania, Philadelphia, PA, USA.

^3^Key Laboratory of Exploration and Utilization of Aquatic Genetic Resources (Ministry of Education), Shanghai Ocean University, Shanghai, China.

^4^Interdisciplinary Research Center on Biology and Chemistry, Shanghai Institute of Organic Chemistry, Chinese Academy of Sciences, Shanghai, 201210, China.

^5^University of the Chinese Academy of Sciences, Beijing, 100049, China.

^6^Department of Pathology and Laboratory Medicine, Penn Neurodegeneration Genomics Center, Perelman School of Medicine at the University of Pennsylvania, PA, USA.

^7^Geriatric Research Education and Clinical Center, Veterans Affairs Puget Sound

Health Care System, Seattle, WA 98108, USA.

^8^Department of Psychiatry and Behavioral

Sciences, University of Washington School of Medicine, Seattle, WA 98195, USA.

^9^Division of Gerontology and Geriatric Medicine, Department of Medicine, University

of Washington School of Medicine, Seattle, WA 98104, USA.

^10^Translational Neuropathology Research Laboratory, Department of Pathology and Laboratory Medicine, Perelman School of Medicine at the University of Pennsylvania, PA, USA.

**†** Deceased on February 8^th^, 2022

* Correspondence: Hong Xu (hongxu@upenn.edu) or Virginia M.-Y. Lee (vmylee@upenn.edu)

**Sup. Table 1 Antibody used in the study.**

| **Antibody Name** | **Specificity** | **Host Species/IgG types** | **Dilutions** | **Source** |
| --- | --- | --- | --- | --- |
| AT8 | p-tau (phosphorylated at Ser202 and Thr 205) | mouse monoclonal, IgG1 | 1:1000 (WB); 1:10000 (IHC). | Thermo Scientific, MN1020 |
| PHF-1 | p-tau (phosphorylated at Ser396 and Ser404) | mouse monoclonal, IgG1 | 1:1000 (WB) | Gift from Dr. Peter Davies |
| AT180 | p-tau (phosphorylated at Thr231) | mouse monoclonal, IgG1 | 1:1000 (IHC) | Thermo Scientific, MN1040 |
| 81A | p-α-Synuclein (phosphorylated at Ser129) | mouse monoclonal, IgG2a | 1:1000 (ICC) | Millipore, MABN826 |
| GFAP | GFAP | rabbit polyclonal | 1:2000 (IHC), 1:1000 (WB) | Proteintech, 16825-1-AP |
| Anti-Iba1 | IBA1 | rabbit polyclonal | 1:1000 (IHC), 1:1000 (WB) | Wako #019-19741 |
| Anti-Sox10 | Human Sox10 | goat polyclonal | 1:3000 (IF) | R&D Systems, AF2864 |
| Anti-MSUT2 | MSUT2 | rabbit polyclonal | 1: 2000 (IHC) Abc kit no AR | Novus Biology, NBP13537 |
| R2295M | mouse tau | rabbit polyclonal | 1: 2000 (ICC), 1:2000 (WB) | Home made |
| T49 | mouse tau | mouse monoclonal, IgG1 | 1: 1000 (ICC), 1:1000 (WB) | Home made |
| Anti-NeuN | Fox-3 | mouse monoclonal, IgG1 | 1: 1000 (WB); 1:2000 (IF) | Millipore, MAB377 |
| H31L21 | amyloid precusor protein 707-713aa | rabbit polyclonal | 1: 3000 (IHC) | Thermo Scientific, 700254 |
| 17028 | MAP2 | rabbit polyclonal | 1: 1000 (ICC), 1:1000 (WB) | Home made |
| Anti-TUBB3 | beta-Tubulin | mouse monoclonal, IgG2a | 1: 1000 (WB); 1:5000 (ICC) | BioLegend, 801202 |
| 22C11 | APP N-terminal (aa66-81) | mouse monoclonal, IgG1 | 1:4000 (IHC); 1:2000 (WB) | Millipore, MAB348 |
| 17025 | total tau | rabbit polyclonal | 1: 1000 (WB) | Home made |
| Anti-Synapsin I | synapsin 1a and 1b | rabbit polyclonal | 1: 2000 (WB) | Millipore, AB1543P |
| Anti-Synaptophysin | synaptophysin | mouse monoclonal, IgG1 | 1: 2000 (WB) | Millipore, MAB368 |
| Anti-PSD95 | postsynaptic Density-95 | mouse monoclonal, IgG2a | 1: 2000 (WB) | Thermo Scientific, MA1-046 |
| Anti-K48-polyUb | K48-linkage Specific Polyubiquitin | rabbit polyclonal | 1:2000 (WB) | Cell Signaling Technology Inc., 4289S |
| Anti-Ubiquitin | ubiquitin | mouse monoclonal, IgG1k | 1: 1000 (WB) | Millipore, MAB1510 |
| EP1536Y | pS129-α-synuclein | rabbit monoclonal | 1:5000 (IHC) | Abcam, ab51253 |
| Anti-VCP (7F3) | Valosin-containing protein (VCP) | rabbit monoclonal | 1:2000 (WB) | Cell Signaling Technology Inc., 2649 |
| Anti-HSP70 | HSP70 | rabbit polyclonal | 1:2000 (WB) | Cell Signaling Technology Inc., 4872 |
| Anti-GAPDH | Glyceraldehyde-3-Phosphate Dehydrogenase | mouse monoclonal, IgG1 | 1:5000 (WB) | Advanced ImmunoChemical Inc., 2-RGM2 |
| Adenosine A1R Antibody | adenosine receptor 1 | rabbit polyclonal | 1:2000 (WB) | Novus Biology, NB300-549 |

**
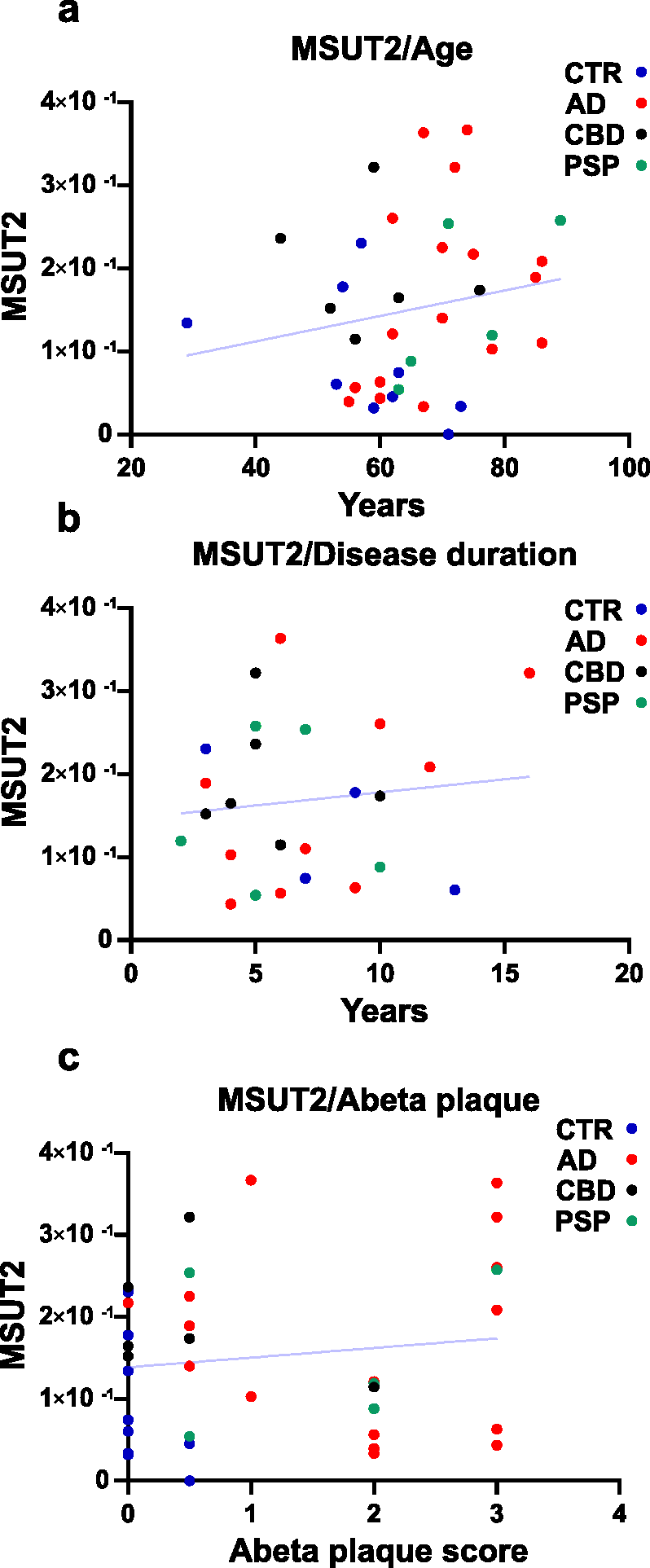
**

**Sup. Fig. 1 MSUT2 expression is not correlated with other pathoclinical features of the disease.**

1. Correlation of MSUT2 immunoreactivity MFI in brain sections from different tauopathies with the age of patients. R^2^ = 0.03479, P = 0.2690 by normal linear regression. Each dot represents one individual case.
2. Correlation of MSUT2 immunoreactivity MFI in brain sections with disease duration from different tauopathy cases. R^2^ = 0.01386, P = 0.5752 by normal linear regression. Each dot represents one individual case.
3. Correlation of MSUT2 immunoreactivity MFI with Abeta plaque burden in brain sections from disease cases. R^2^ = 0.0181, P = 0.4340 by normal linear regression. Each dot represents one individual case.


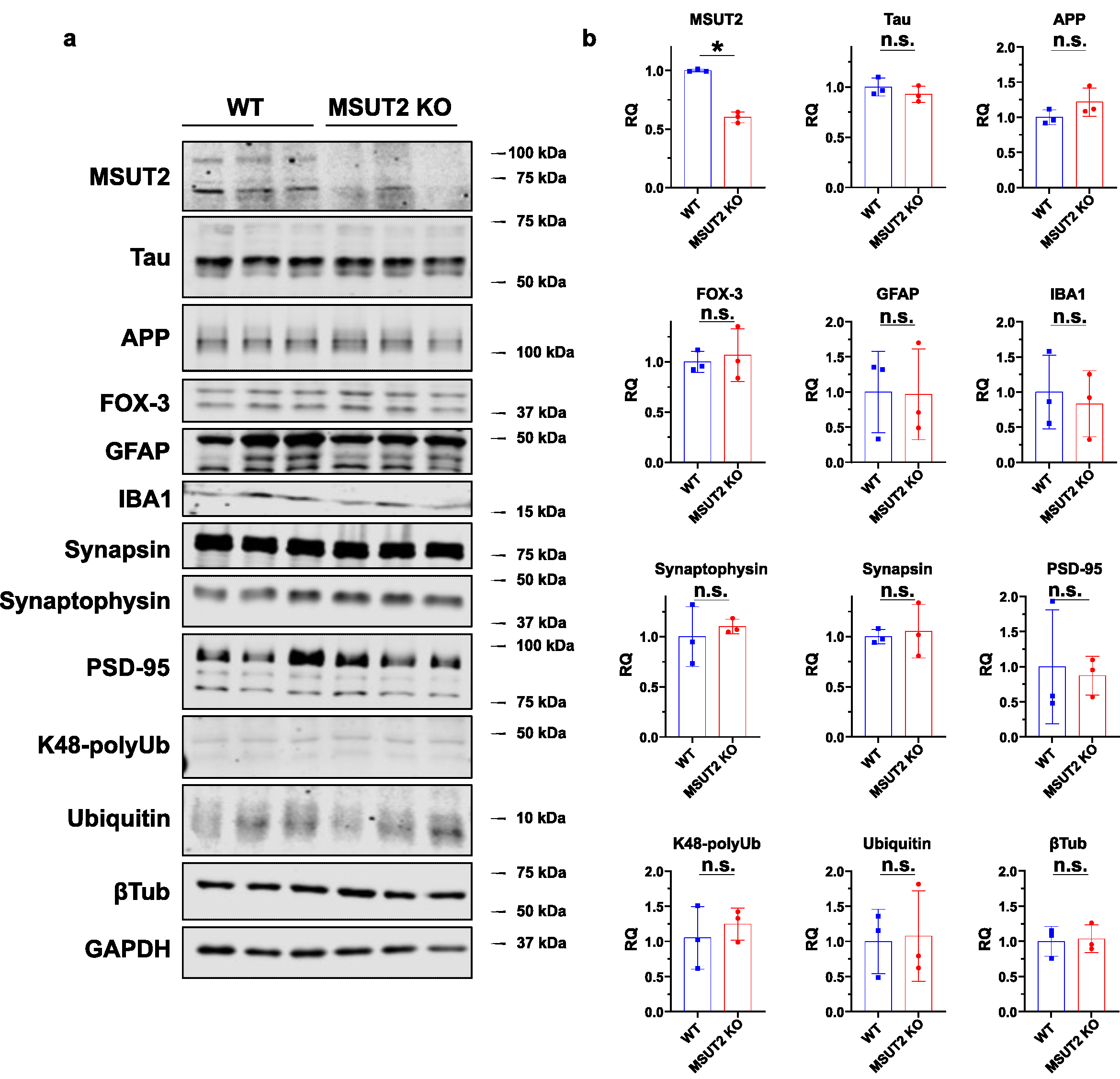


**Sup. Fig. 2 Different protein markers are not significantly changed in MSUT2 KO mice.**

1. Immunoblots of 3-month-old mouse brain lysates probed with MSUT2, 17025 (Tau), 22C11 (APP), NeuN (FOX-3), 2.2 B10 (GFAP), IBA1, Synapsin I, SY38 (Synaptophysin), PSD-95, K48 (K48-polyUb), Ubiquitin, Beta-tubulin (βTub), and GAPDH antibodies. Note that the MSUT2 KO mice are functional KO with the residual expression of inactive MSUT2 protein.
2. Quantification of the optical density from the fluorescent signal in **a**. * P < 0.05, n.s., not significant by t-test, MSUT2 KO (KO) vs. wild-type (WT) mouse, n = 3 mice per group. The error bars represent the standard deviation.


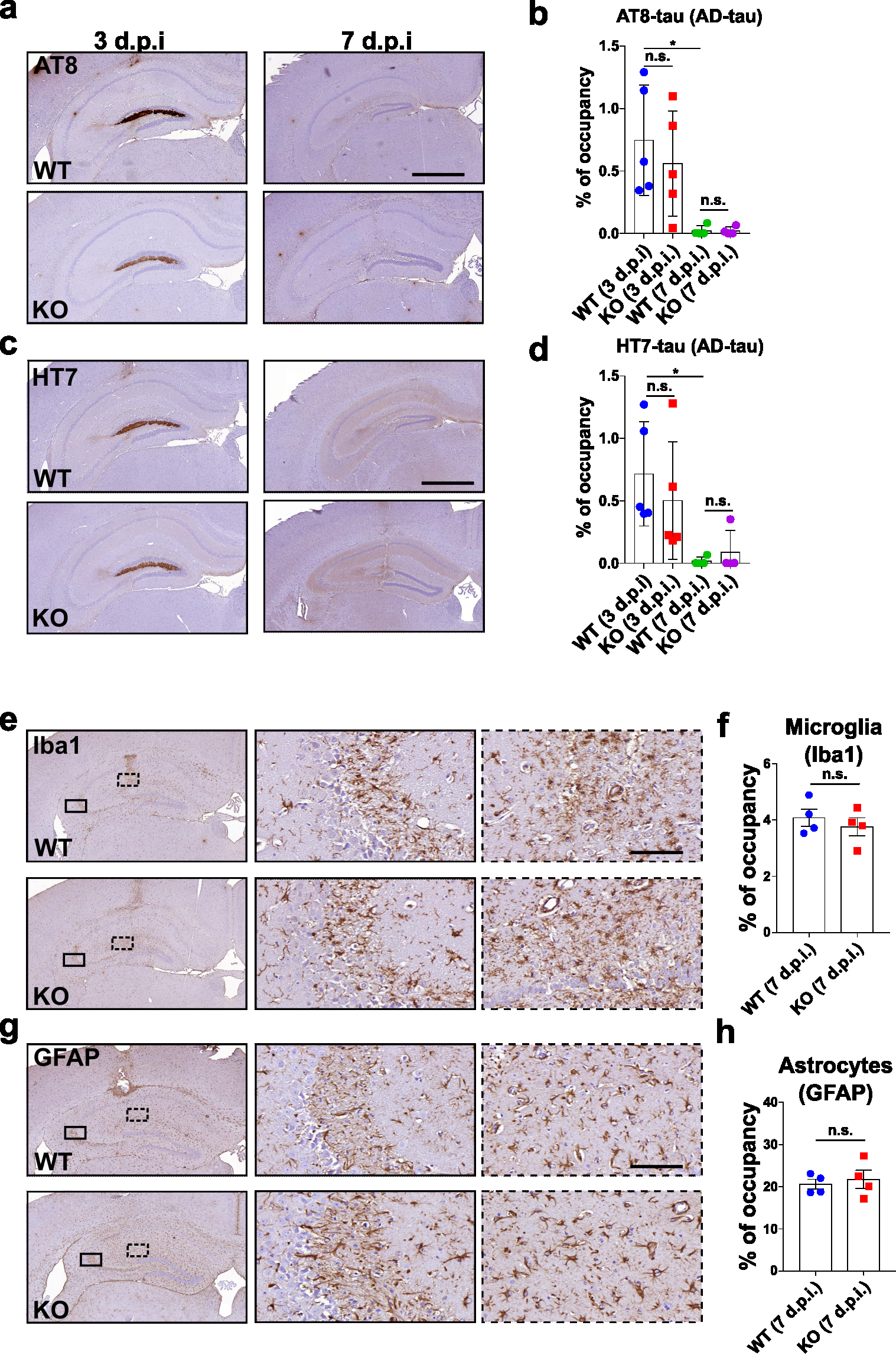


**Sup. Fig. 3 Turnover of injected tau seeds and acute neuroinflammation are not changed in MSUT2 KO mice.**

1. AD-tau-injected WT (wild-type) and KO (MSUT2 knockout) mouse brain sections were immunohistochemically stained with AT8 p-tau antibody at 3 and 7 d.p.i. Scale bar = 250 µm.
2. Quantification of AT8 immunoreactive area from sections obtained from the hippocampal region of AD-tau-injected WT and MSUT2 KO mice at 3 and 7 d.p.i. as in **a**. n.s., not significant, * P < 0.05 by one-way ANOVA followed by Tukey’s post hoc test, MSUT2 KO (KO) vs. wild-type (WT) mouse at each injection time, n = 4-5 mice per group. The error bars represent the standard deviation.
3. AD-tau-injected WT and MSUT2 KO mouse brain sections were immunohistochemically stained with HT7 human-tau antibody at 3 and 7 d.p.i. Scale bar = 250 µm.
4. Quantification of HT7 immunoreactive area from sections obtained from the hippocampal region of AD-tau-injected WT and MSUT2 KO mouse brains at 3 and 7 d.p.i. as in **c**. n.s., not significant, * P < 0.05 by t-test, MSUT2 KO (KO) vs. wild-type (WT) mouse at each injection time, n = 4 per group. The error bars represent the standard deviation.
5. Representative images of AD-tau-injected WT and MSUT2 KO mouse brains sections at 7 d.p.i. stained with Iba1 antibody to visualize microglia. Scale bar = 50 µm.
6. Quantification of Iba1 immunoreactive area in sections from the hippocampal region of AD-tau-injected WT and MSUT2 KO mice at 7 d.p.i. as in **e**. n.s., not significant by t-test, MSUT2 KO (KO) vs. wild-type (WT) mouse, n = 4 mice per group. The error bars represent the standard deviation.
7. Representative images of AD-tau-injected WT and MSUT2 KO mouse brains sections at 7 d.p.i. stained with GFAP antibody to visualize astrocytes. Scale bar = 50 µm.
8. Quantification of GFAP immunoreactive area in sections from the hippocampal region of AD-tau-injected WT and MSUT2 KO at 7 d.p.i., as in **g**. n.s., not significant by t-test, MSUT2 KO (KO) vs. wild-type (WT) mouse, n = 4 mice per group. The error bars represent the standard deviation.


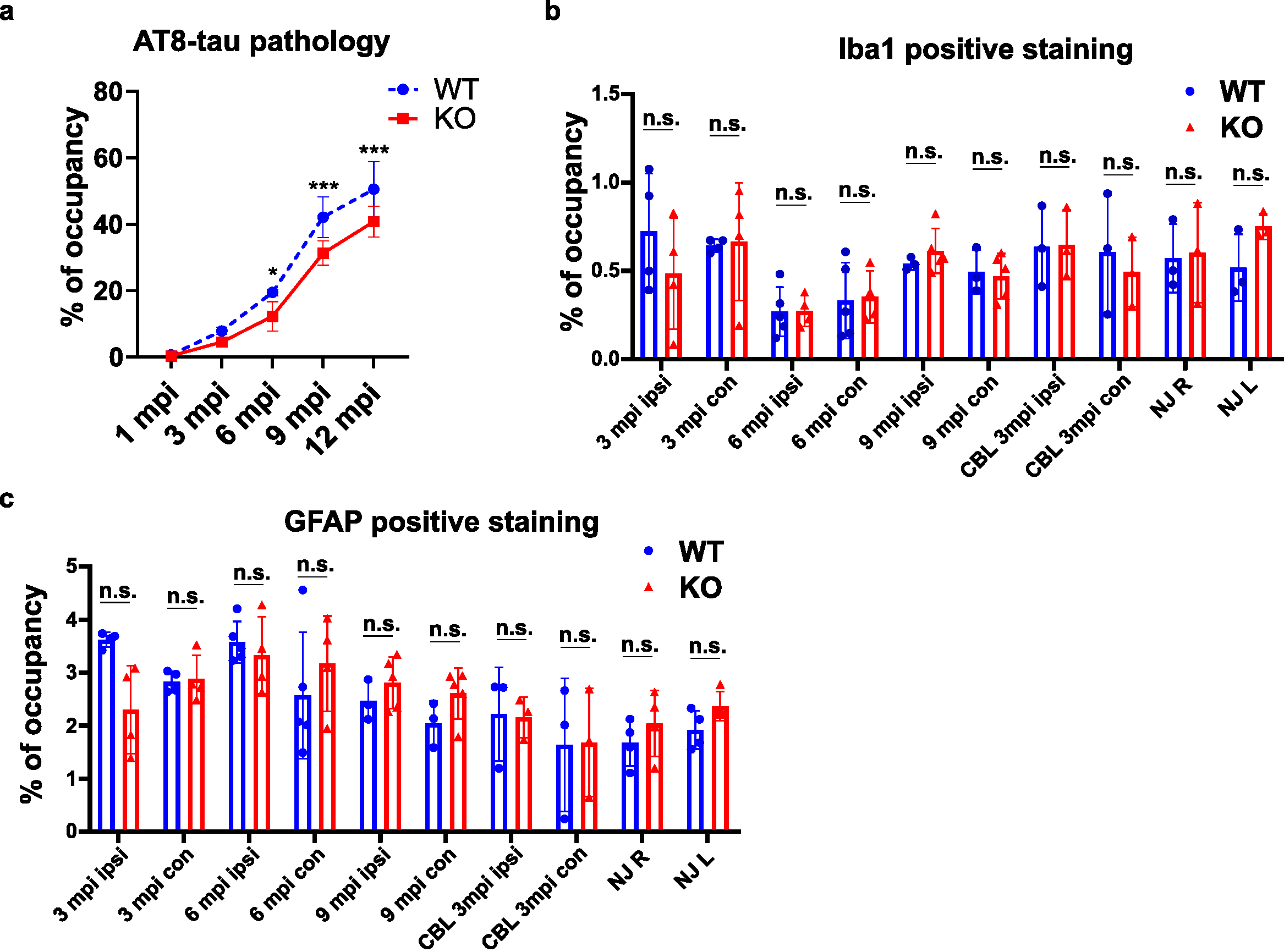


**Sup. Fig. 4 Chronic neuroinflammation does not change with the spreading of tau pathology in MSUT2 KO mice.**

1. Quantification of AT8 immunoreactivity area in the ipsilateral hippocampal regions of AD-tau-injected wild-type (WT) and MSUT2 KO (KO) mice at 1 to 12 m.p.i. * P < 0.05, *** P < 0.001 by two-way ANOVA followed by Bonferroni's post hoc test, MSUT2 KO (KO) vs. wild-type (WT) mouse, n = 6 per group. The error bars represent the standard deviation.
2. Quantification of Iba1 immunoreactive area in AD-tau-injected wild-type (WT) and MSUT2 KO (KO) mouse brains at 3, 6, 9 m.p.i., as well as non-tau control brain lysate (CBL) injected mice and non-injected (NJ) mouse brains. Ispi = ipsilateral injection side; con = contralateral injection side; R, right side, L, left side. n.s., not significant by two-way ANOVA followed by Bonferroni's post hoc test, n = 3-4 mice per group. The error bars represent the standard deviation.
3. Quantification of GFAP immunoreactive area in AD-tau-injected wild-type (WT) and MSUT2 KO (KO) mouse brains at 3, 6, and 9 m.p.i., as well as non-tau control brain lysate (CBL) injected mice and non-injected (NJ) mouse brains. Ispi = ipsilateral injection side; con = contralateral injection side; R, right side; L, left side. n.s., not significant by two-way ANOVA followed by Bonferroni's post hoc test, WT vs. KO, n = 3-4 mice per group. The error bars represent the standard deviation.


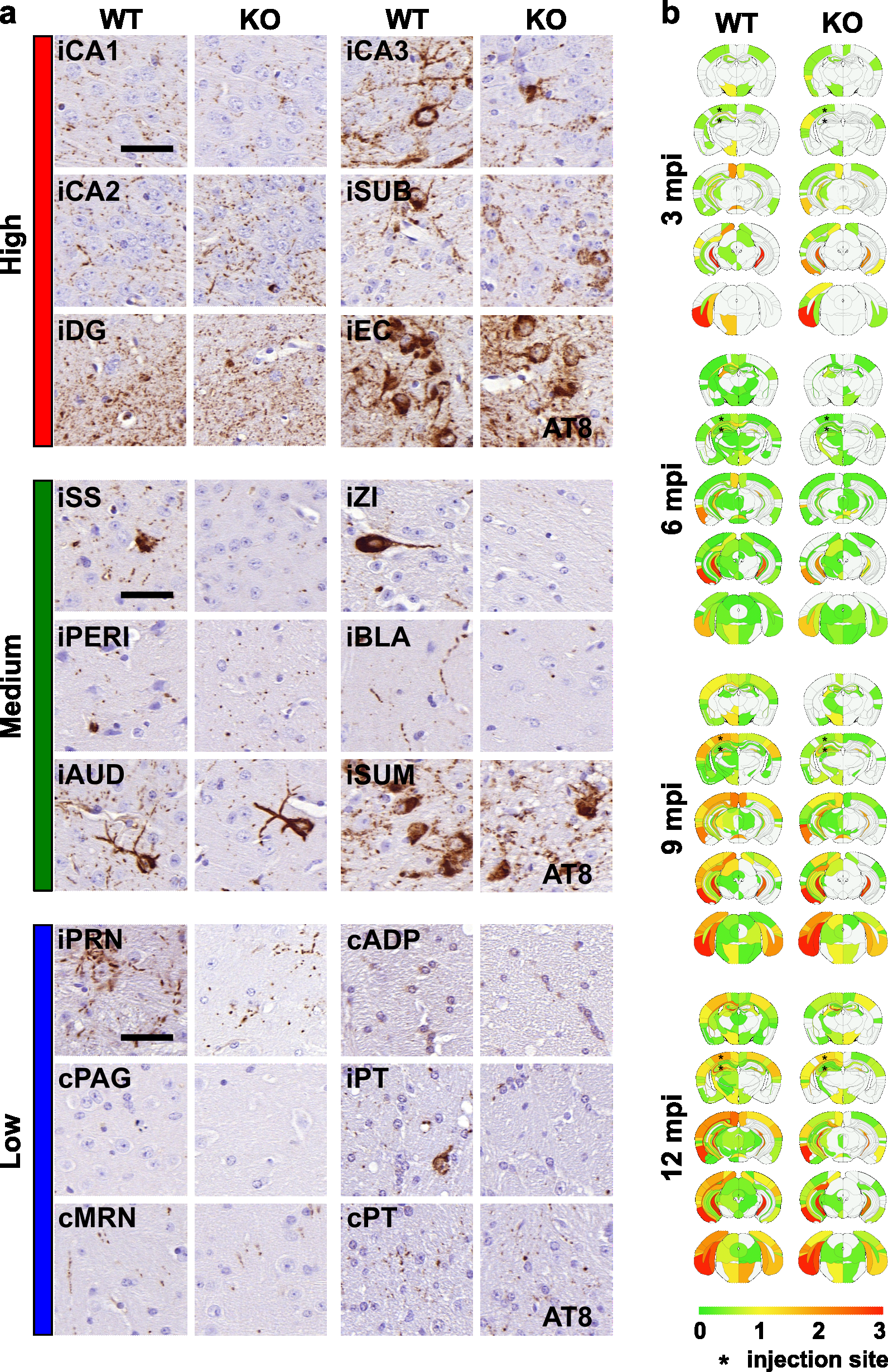


**Sup. Fig. 5 The brain region distribution of tau pathology differs in AD-tau-injected MSUT2 KO and wild-type littermate mouse brains.**

1. Representative images of AT8-positive tau pathology in AD-tau-injected MSUT2 KO (KO) and wild-type (WT) mouse brain regions at 12 m.p.i., with high (red), medium (green), low (blue) anterograde connectivity strength to the injection sites (dorsal hippocampus and cortex). i = ipsilateral; c = contralateral; CA = cornu ammonis; SUB = subiculum; DG = dentate gyrus; EC = entorhinal cortex; SS = somatosensory areas; ZI = zona incerta; PERI = perirhinal area; BLA = basolateral amygdalar nucleus; AUD = auditory areas; SUM = supramammillary nucleus; PRN = pontine reticular nucleus; ADP = anterodorsal preoptic nucleus; PAG = periaqueductal gray; PT = parataenial nucleus; MRN = midbrain reticular nucleus. Scale bar = 10 µm.
2. Semi-quantitative assessment of tau pathology in different brain regions of AD-tau-injected MSUT2 KO (KO) and wild-type littermates (WT) at 3, 6, 9, 12 m.p.i. Numeric scores were given based on the abundance of AT8-positive tau pathology: 0 = none; 1 = little; 2 = moderate; 3 = abundant. Heatmap shows distribution and abundance of tau pathology. Color hue indicates the relative abundance of tau pathology. n = 3 per group.


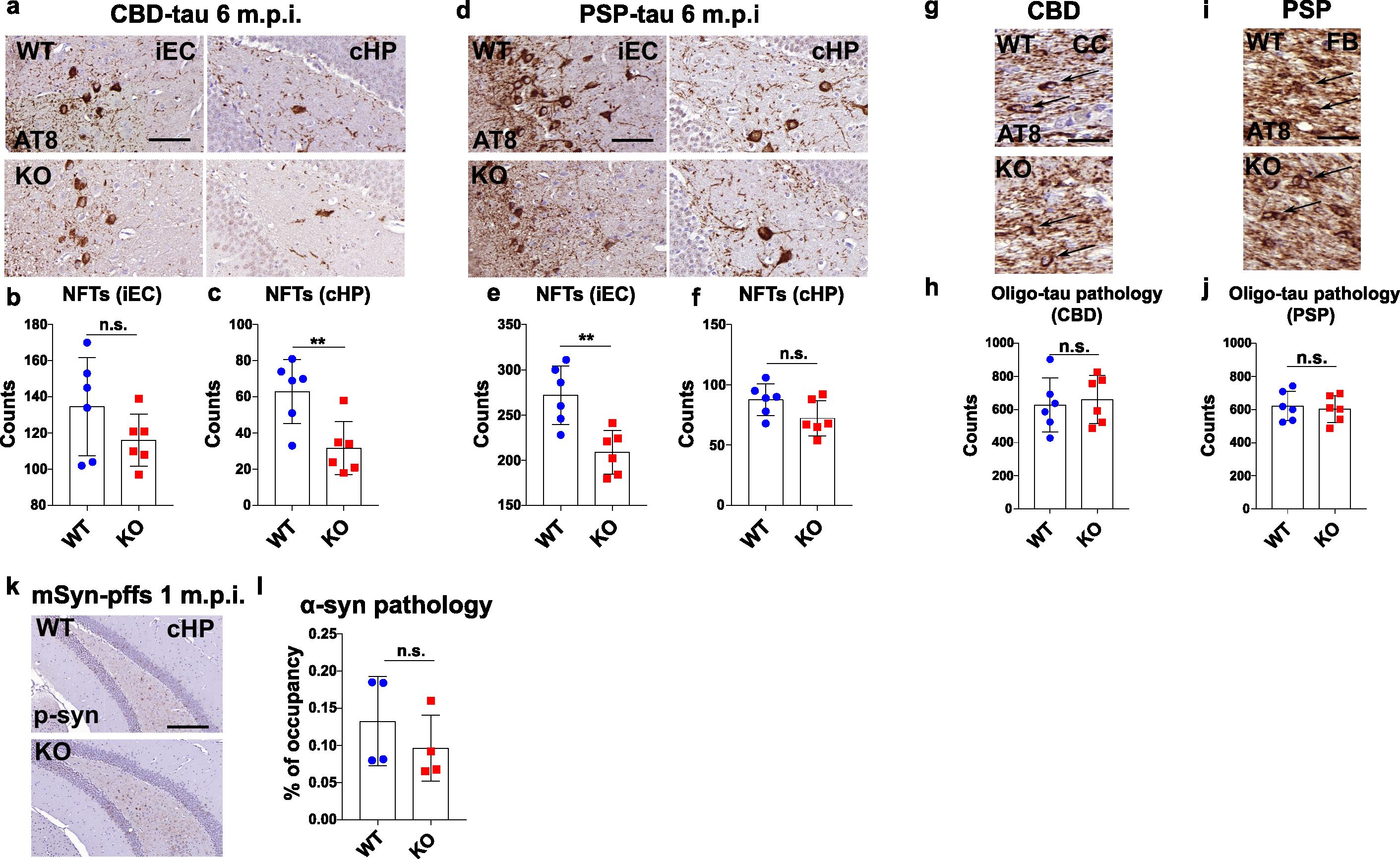


**Sup. Fig. 6 Tau pathology from additional brain regions of CBD-tau, PSP-tau, or mSyn-pffs-injected mice.**

1. Representative images of tau pathology in the ipsilateral entorhinal cortical (iEC) and contralateral hippocampal (cHP) regions of CBD-tau-injected MSUT2 KO (KO) and wild-type (WT) mouse brains. Brain sections were stained with AT8 antibody for tau pathology at 6 m.p.i. NFTs = neurofibrillary tangles. Scale bar = 50 μm.
2. **c.** Quantification of AT8-positive NFT counts in the ipsilateral entorhinal cortical (iEC) and contralateral hippocampal (cHP) regions of CBD-tau-injected MSUT2 KO (KO) and wild-type (WT) mouse brains in sections stained as in **a**. ** P < 0.01, n.s., not significant by t-test, MSUT2 KO (KO) vs. wild-type (WT) mouse, n = 6 mice per group. Error bars represent the standard deviation.
3. Representative images of tau pathology in the ipsilateral entorhinal cortical (iEC) and contralateral hippocampal (cHP) regions of PSP-tau-injected MSUT2 KO (KO) and wild-type (WT) mouse brains. Brain sections were stained with AT8 antibody for tau pathology at 6 m.p.i. NFTs, neurofibrillary tangles. Scale bar = 50 μm.
4. **f.** Quantification of AT8-positive NFT counts in the ipsilateral entorhinal cortical (iEC) and contralateral hippocampal (cHP) regions of PSP-tau-injected MSUT2 KO (KO) and wild-type (WT) mouse brains in sections stained as in **d**. ** P < 0.01, n.s., not significant by t-test, MSUT2 KO (KO) vs. wild-type (WT) mouse, n = 6 mice per group. Error bars represent the standard deviation.
5. **i**. Representative images of oligodendroglia tau pathology in the corpus callosum (CC) region of CBD-tau-injected and fimbria (FB) region of PSP-tau injected MSUT2 KO (KO) and wild-type (WT) mouse brains. Brain sections were stained with AT8 antibody for tau pathology at 6 m.p.i. Arrows highlighted oligodendroglia tau pathology. Scale bar = 10 μm.
6. **j.** Quantification of oligodendroglia tau pathology in **g** and **i**. n.s., not significant by t-test, MSUT2 KO (KO) vs. wild-type (WT) mouse, n = 6 mice per group. Error bars represent the standard deviation.
7. Representative images showing α-synuclein pathology in contralateral hippocampal (cHP) region of mSyn-pff-injected MSUT2 KO (KO) and wild-type (WT) mice. Mouse brain sections were stained with EP1536P antibody to reveal α-synuclein pathology. Scale bar = 200 μm.
8. Quantification of α-synuclein pathology area in the contralateral hippocampal regions of mSyn-pffs-injected mice in sections stained as in **g**. n.s., not significant by t-test, MSUT2 KO (KO) vs. wild-type (WT), n = 4 mice per group. Error bars represent the standard deviation.


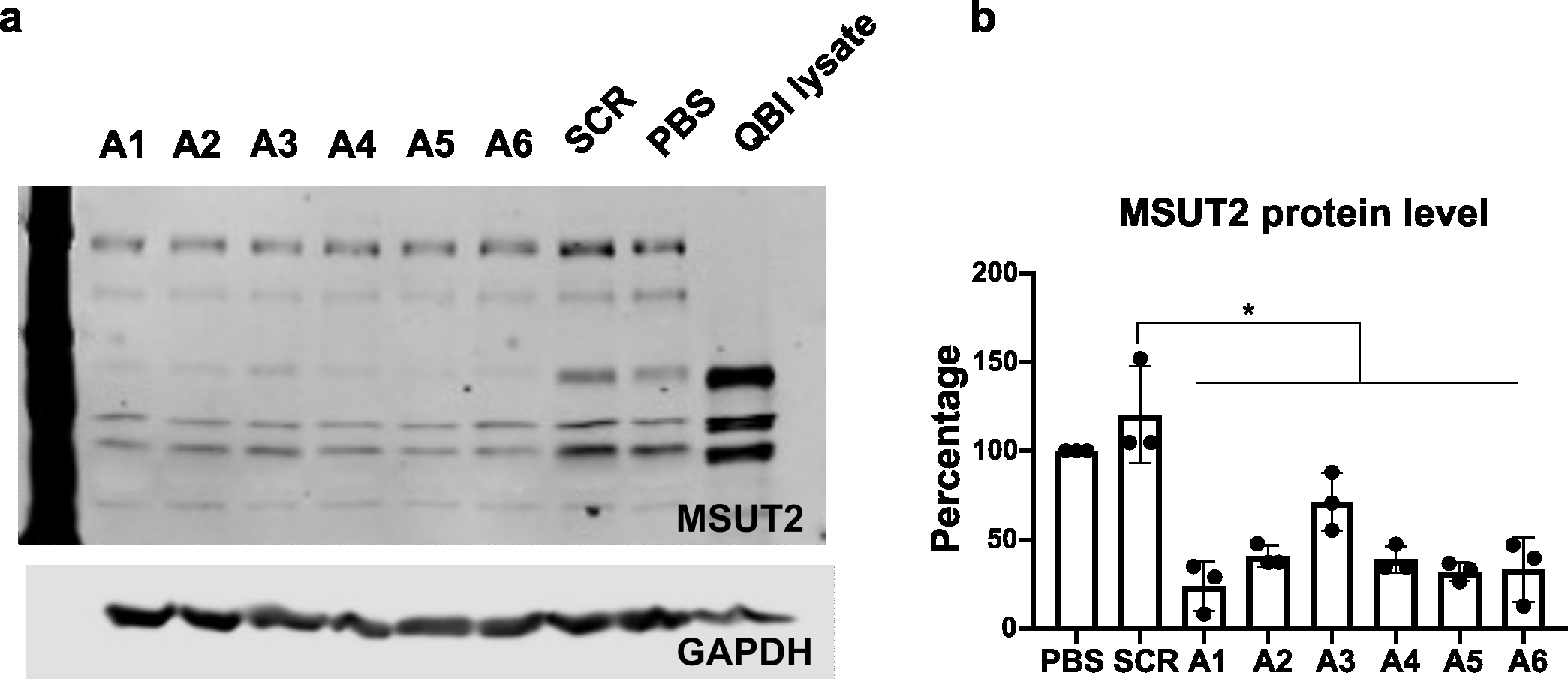


**Sup. Fig. 7 Antisense oligonucleotides (ASOs) efficiently knockdown MSUT2 expression in primary neurons.**

1. Representative image showing immunoblot of primary neuron and QBI HEK293a cell lysates probed with MSUT2 or GAPDH (loading control) antibodies. Primary neurons were treated with PBS or ASOs (against mouse *Msut2* A1-A6 or SCR control) at DIV2 and harvested at DIV21. Untreated QBI HEK293a cell lysate was used as the positive control for the MSUT2 protein.
2. Quantification of MSUT2 and GAPDH optical density from immunoblots as shown in **a**. Data were normalized to GAPDH in each sample. Signals in PBS-treated samples were set as 100%. * P < 0.05 by one-way ANOVA followed by Tukey’s post hoc test, n = 3 biological repeats. Error bars represent the standard deviation.


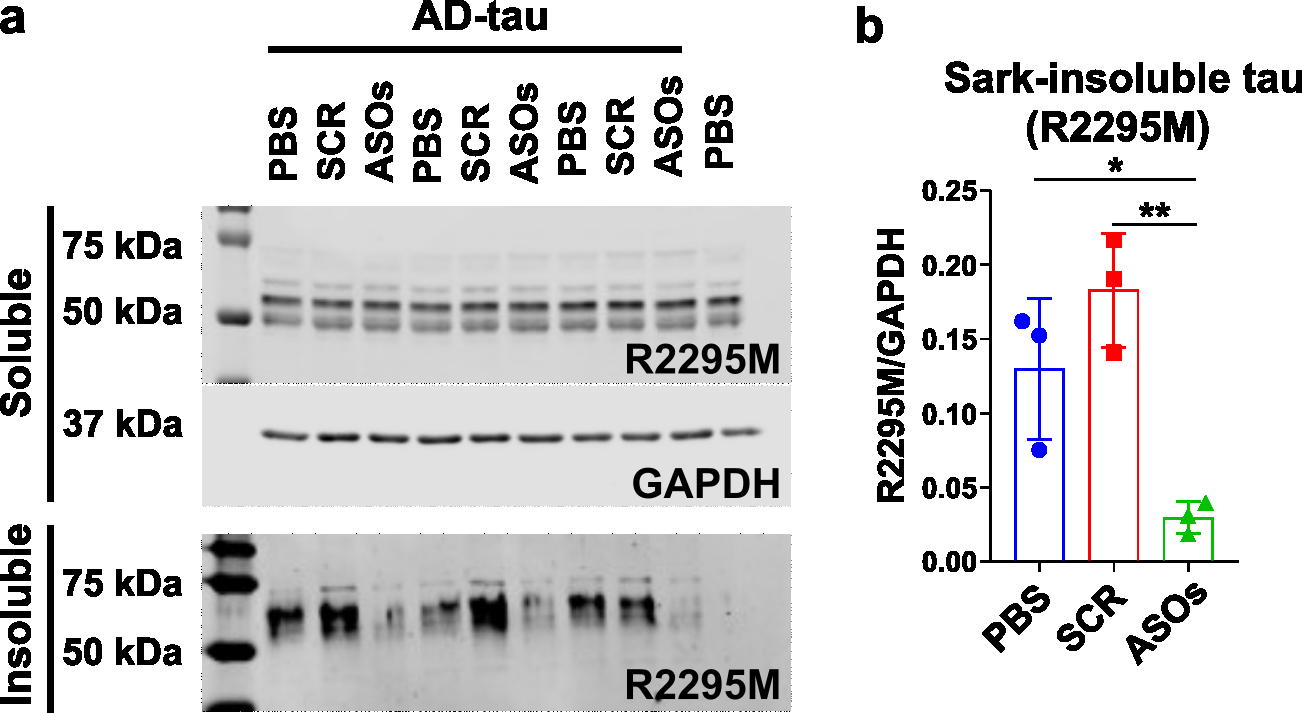


**Sup. Fig. 8 Insoluble tau was reduced in AD-tau seeded neurons treated MSUT2 ASOs.**

1. Primary neurons were treated with ASOs against MSUT2 (ASOs), scrambled ASO control (SCR) or PBS at DIV2, followed by AD-tau addition at DIV7. Cell lysates were harvested at DIV21 and fractionated into sarkosyl-soluble and -insoluble fractions and immunoreactive protein bands were resolved with immunoblots probed with R2295M (tau) and GAPDH antibodies.
2. Quantification of R2295M and GAPDH optical density in immunoblot analyses as depicted in **a**. The optical density of R2295M was normalized to that of GAPDH. * P < 0.05, ** P < 0.01 by one-way ANOVA followed by Tukey's multiple comparisons test, n = 3 biological repeats per group. Error bars represent the standard deviation.


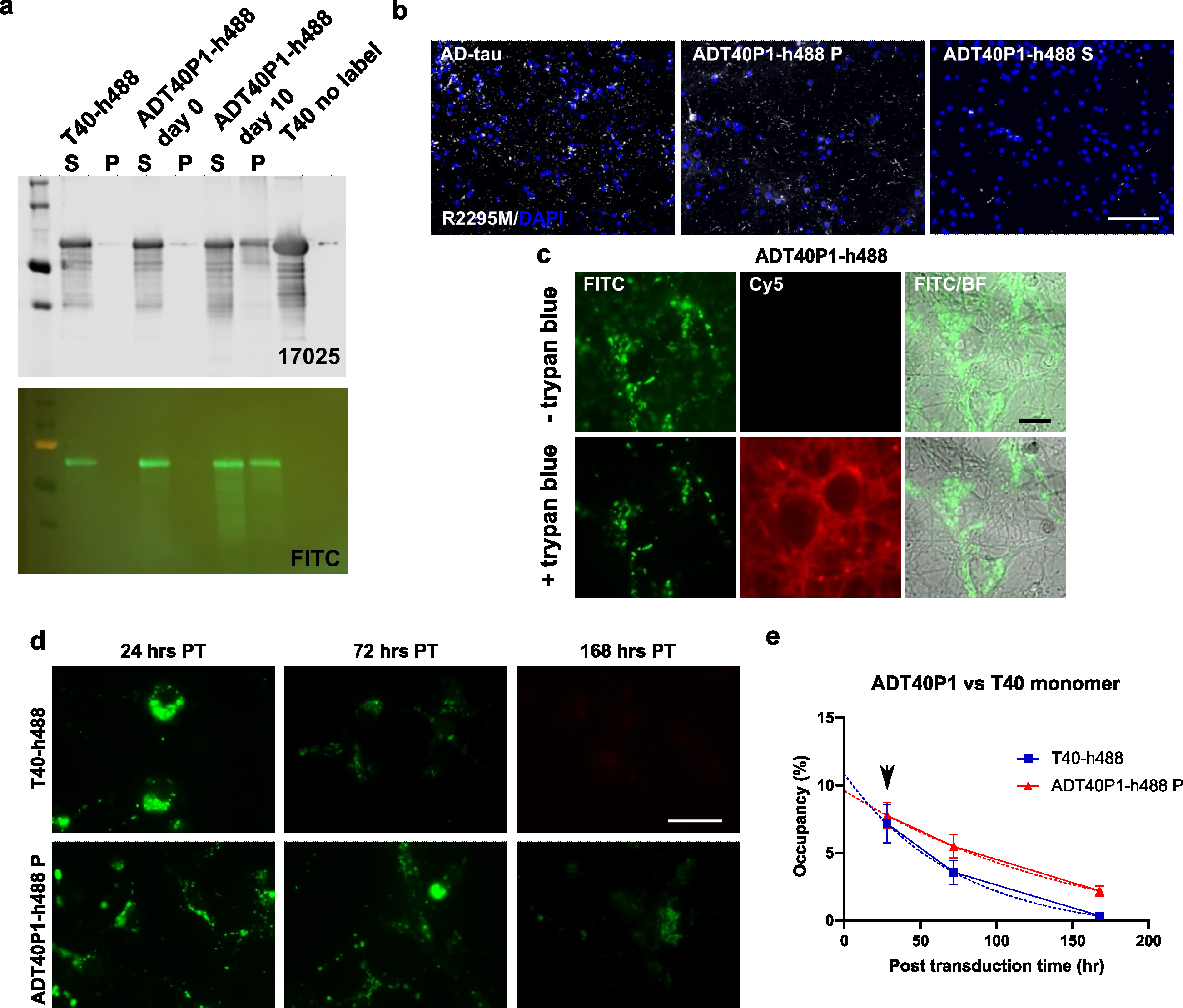


**Sup.** **Fig. 9 Fluorescently-labeled ADT40P1 is pathogenic in primary neurons.**

1. Immunoblot showing supernatant (S) and pellet (P) fractions after centrifugation of non-seeded monomers of recombinant T40 tau labeled with hylite488 dye (T40-h488), and AD-tau-seeded T40-h488 monomer before (ADT40P1-h488 day 0) and after (ADT40P1-h488 day 10) the seeding reaction. T40 monomer was loaded for reference. FITC channel shows the fluorescent signal of Hylite 488 dye. Total tau was detected with the 17025 antibody.
2. Immunocytochemistry showing tau pathology in mouse primary neurons treated with AD-tau or amplified ADT40P1-h488. ADT40P1-h488 was fractionated into soluble (supernatant) and insoluble (pellet) fractions after centrifugation at 100,000 g for 30 minutes. Primary neurons were treated with the same dose (10 ng/100,000 cells) of AD-tau, or pellet (P), or supernatant (S) fractions of amplified ADT40P1-h488. Cells were stained with R2295M antibody to detect mouse tau pathology and DAPI for nuclei. Scale bar = 100 μm.
3. Representative images showing live images of primary neurons treated with fluorescently labeled ADT40P1-h488 before and after trypan blue quenching. Primary neurons were treated with ADT40P1-h488 (10 ng/100,000 cells) for 4 hrs and the medium was changed with that containing trypan blue (500 µM) to quench the extracellular hylite488 signal. Live cell images were taken before and after the medium change. The FITC channel shows fluorescent signals from hylite488. Cy5 channel shows fluorescent signals from trypan blue. BF = bright field. Scale bar = 5 μm.
4. Representative images of mouse primary neurons treated with ADT40P1-h488 at different time points. Mouse primary neurons were treated with hylite488-labeled T40 monomer or the pellet fraction of ADT40P1-h488 at DIV7. The medium was replaced with fresh medium at 24 hrs post-treatment. Live images were immediately taken at 24, 72 and 168 hrs after the addition of trypan blue. Scale bar = 10 µm.
5. Quantification of the intracellular fluorescent area of ADT40P1-488 in images as depicted in **d**. The arrow indicates the time point of medium change. Half-life: T40 monomer = 51.5 hrs, ADT40P1-h488 = 111.6 hrs, non-linear fit. n = 3 biological repeats. Error bars represent the standard error of the mean.


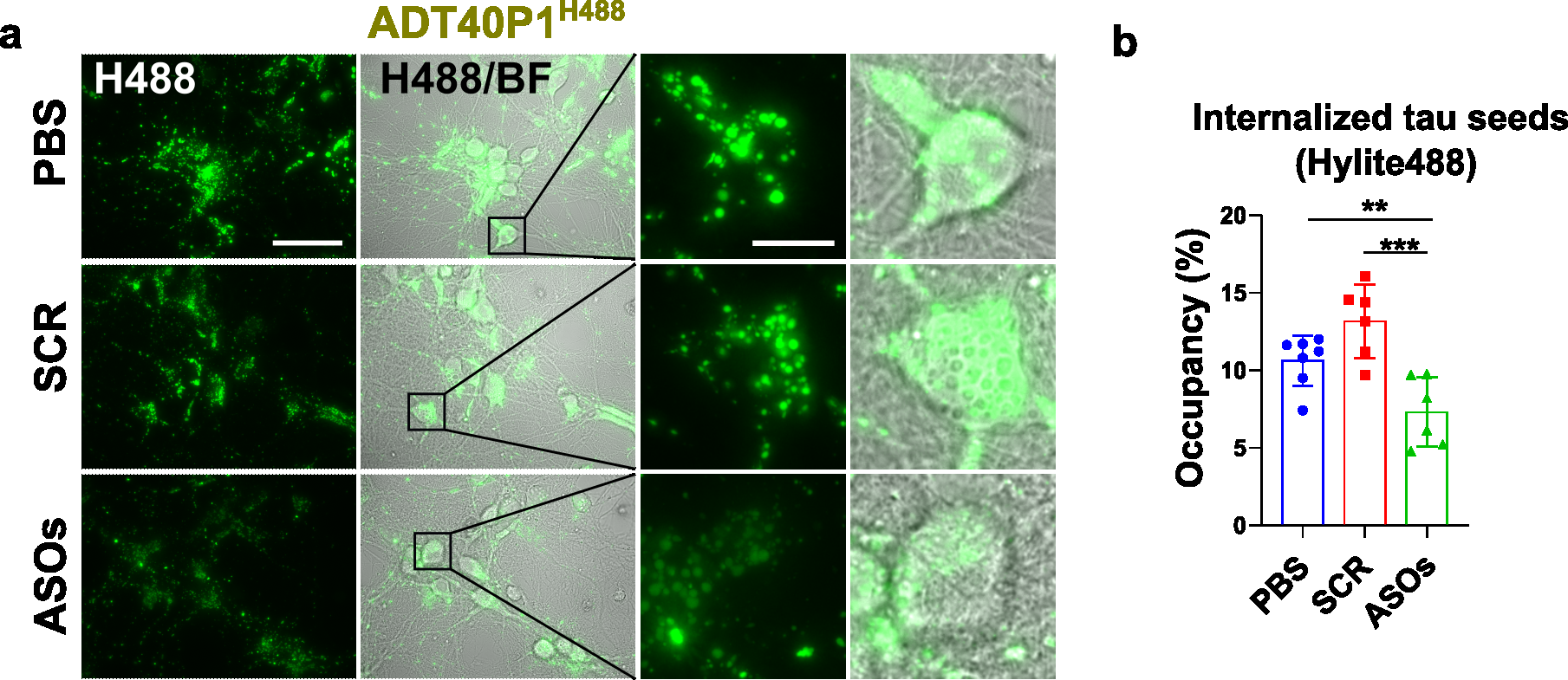


**Sup.** **Fig. 10 Uptake of hylite-labeled ADT40P1 is reduced in MSUT2 KD primary neurons.**

1. Representative images show intracellular fluorescently labeled tau seeds in primary neurons. Neurons were treated with PBS, scrambled control ASOs (SCR), or ASOs against MSUT2 (ASOs) at DIV 2 and hylite 488 dye-labeled AD-tau seeds (ADT40P1-h488) at DIV 6. Live imaging microscopy was performed at DIV 7 (1 d.p.t.). The extracellular signal was quenched with trypan blue (500 µM) before live imaging. BF = bright field. Scale bar = 25 µm (overview) and 5 µm (insets).
2. Quantification of fluorescent signal (fluorescence density x area/neuron counts) in each condition represented in **a**. PBS-treated samples are set as 100%. ** P < 0.01, *** P < 0.001 by one-way ANOVA followed by Tukey's multiple comparisons test, PBS vs. SCR vs. ASOs, n = 6 biological repeats per group. Error bars represent the standard deviation.


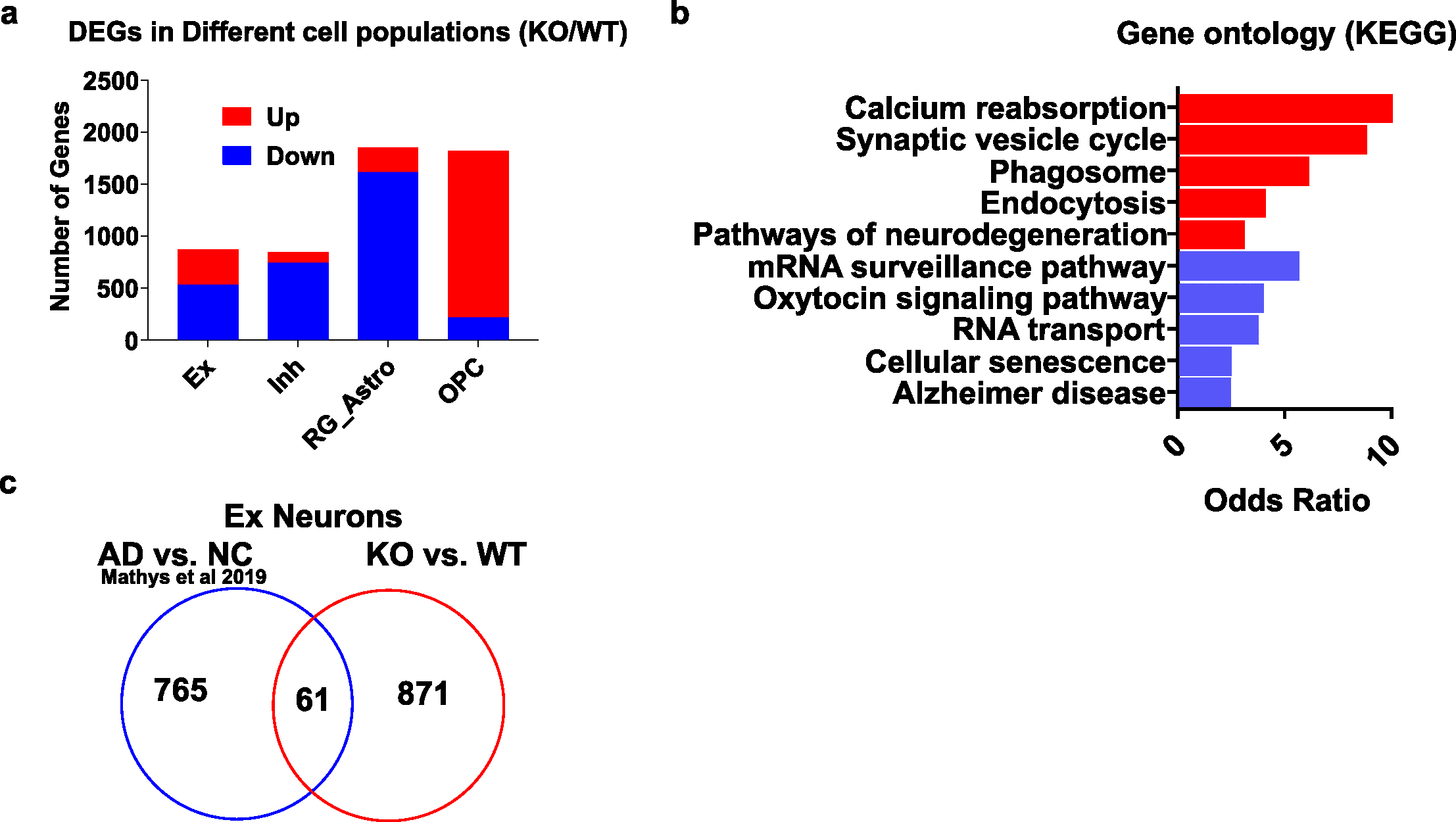


**Sup.** **Fig. 11 More descriptive data are shown for scNT-sequencing.**

1. Number of differentially expressed genes (DEGs) in major cell populations. Upregulated genes are shown in red while downregulated in blue. Ex = excitatory neurons, Inh = inhibitory neurons, MG = microglia, OPC = oligodendrocyte precursor cells.
2. Gene ontology of DEGs in excitatory neurons. The Kyoto Encyclopedia of Genes and Genomes (KEGG) database was used to predict the pathways of DEGs in excitatory neurons. Red represents pathways that are up-regulated, and blue indicates pathways that are down-regulated in MSUT2 KO vs. WT neurons.
3. Overlapping of DEGs in MSUT2 KO excitatory neurons compared to a previously published single-cell sequencing database in human AD patients (DEGs in excitatory neurons) [55].


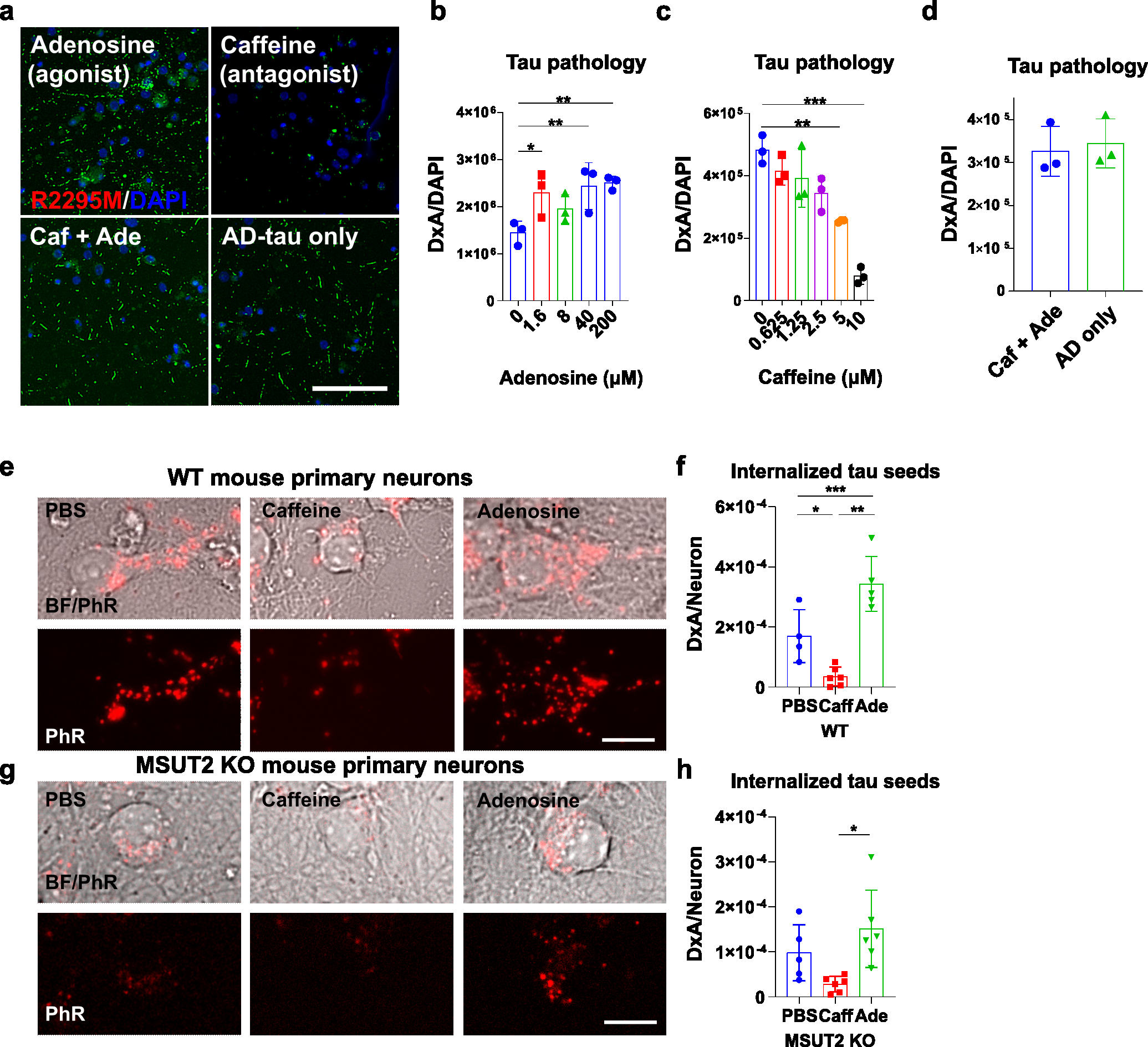


**Sup.** **Fig. 12 Interruption of adenosinergic signaling pathways reduces AD-tau seeded tau pathology in primary neurons.**

1. Representative pictures showing AD-tau-induced tau pathology in neurons treated with adenosine receptor-directed compounds. Wild-type primary neurons were treated with an adenosine receptor agonist (adenosine, 40 μM) and/or antagonist (caffeine, 10 μM) at DIV6, followed by the addition of AD-tau at DIV7. After removing soluble proteins, cells were immunocytochemically stained for aggregated mouse tau with R2295M antibody (in green). DAPI staining reveals cell nuclei (in blue). Scale bar = 125 µm.
2. **c. d.** Quantification of R2295M immunoreactivity in neurons as depicted in **a**. Density * Area normalized to DAPI count (DxA/DAPI). * P < 0.05, ** P < 0.01, *** P < 0.001 by t-test or one-way ANOVA followed by Tukey's multiple comparison, n = 3 biological repeats/condition. Error bars represent the standard deviation.
3. **g.** Representative images showing internalized pathogenic tau seeds in wild type (WT), or MSUT2 KO (MSUT2KO) mouse primary neurons treated with PBS, caffeine, or adenosine, followed by the addition of pHrodo red (PhR)-labeled ADT40P1. Internalized tau seeds were imaged with live cell microscopy 24 hrs post-treatment. BF = Bright field. Scale bar = 5 µm.
4. **h.** Quantification of internalized tau seeds using pHrodo red signal as depicted in **e** and **g**. Fluorescence density x Area normalized to Neuron count (DxA/Neuron). * P < 0.05, ** P < 0.01, *** P < 0.001 by one-way ANOVA followed by Tukey's multiple comparison, n = 4 to 5 biological repeats/condition. Error bars represent the standard deviation.


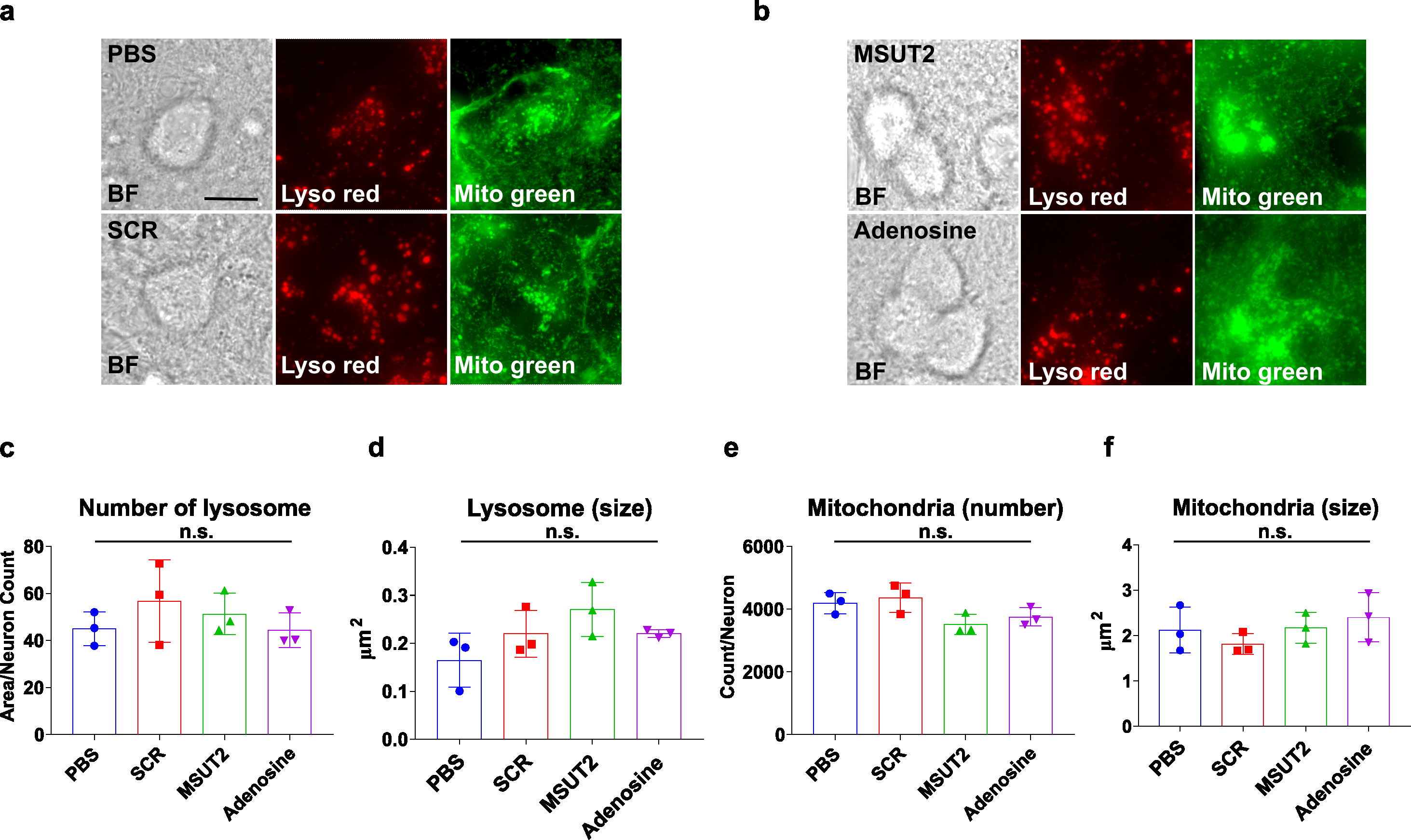


**Sup.** **Fig. 13 Neuronal lysosomes and mitochondria were not significantly affected by MSUT2 or adenosine.**

1. Representative images showing lysosome and mitochondria in primary neurons treated with PBS, scrambled control ASO (SCR), ASOs (MSUT2), or Adenosine at DIV5. Lysotracker (Lyso red) and mitotracker (Mito green) were added at DIV 7 and cells were imaged 30 min later. BF = bright field. Scale bar = 5 µm.
2. **c. d. e.** Quantification of lysotracker and mitotracker signals in studies depicted in **a**. n.s, not significant by one-way ANOVA followed by Tukey's post hoc test, n = 3 biological repeats. Error bars represent the standard deviation.


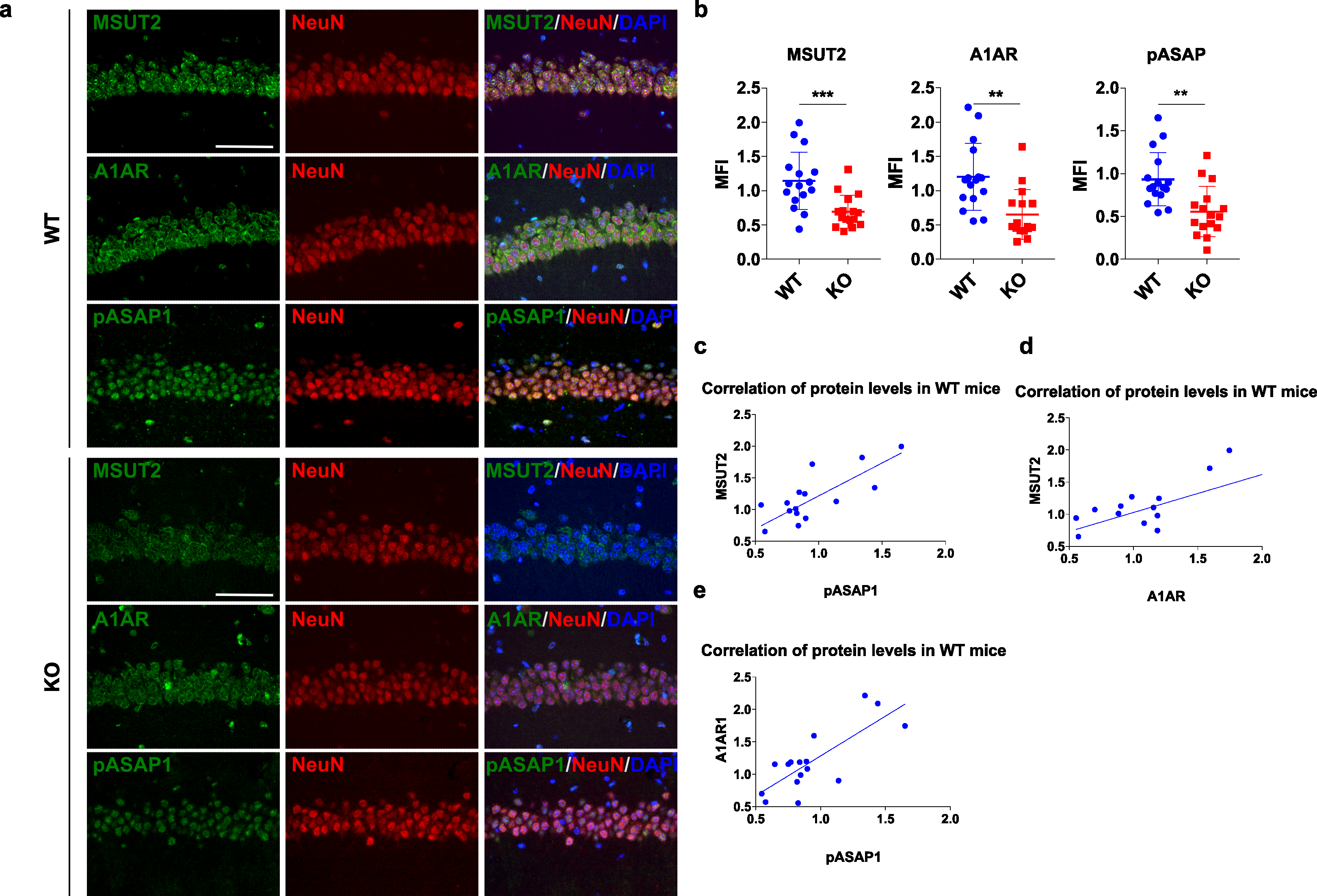


**Sup.** **Fig. 14 Activated ASAP1 is decreased in MSUT2 KO mice.**

1. Representative images showing immunofluorescent co-stainings of MSUT2/NeuN/DAPI, A1AR/NeuN/DAPI, pASAP1/NeuN/DAPI on MSUT2 KO mice (KO) and wild type littermates (WT) in CA1 region. Scale bar = 100 µm.
2. Quantification of the immunoreactivity of MSUT2, A1AR, and pASAP1 in **a**. Protein levels were presented as the mean fluorescent intensity (MFI) of each antibody. ** P < 0.01, *** P < 0.001 by t-test, n = 4 mice per group.
3. **d. e.** Correlations of MSUT2, A1AR, and pASAP1 expression levels in wild type mice in **a**. MSUT2 vs pASAP, P = 0.0005, R^2^ = 0.5898; MSUT2 vs A1AR, P = 0.0031, R^2^ = 0.4752; A1AR vs pASAP1, P = 0.0004, R^2^ = 0.6064 by linear regression.


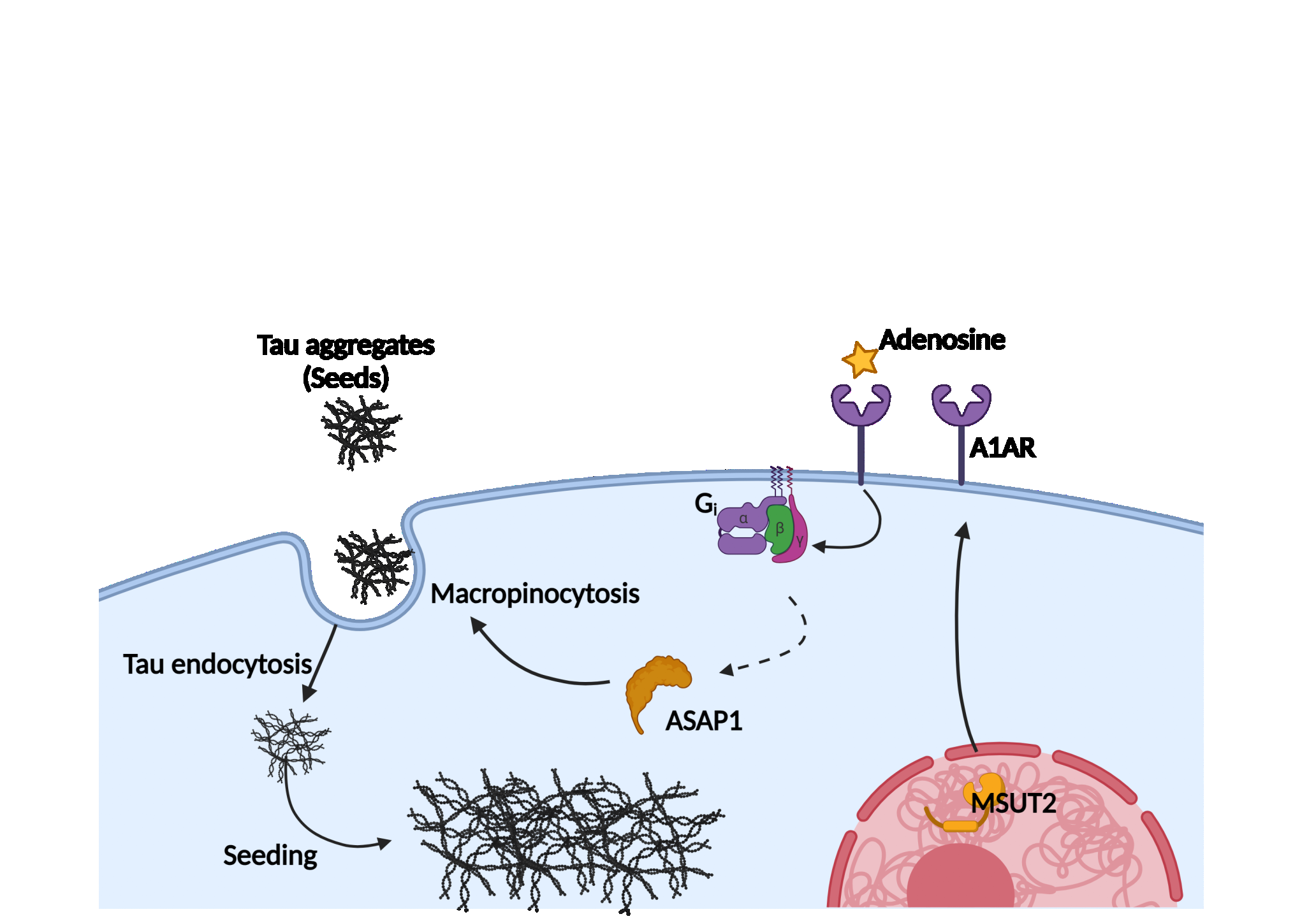


Created with BioRender.com

**Sup. Fig. 15 Schematic image of study findings.**

MSUT2 modulates the stability of A1AR mRNA and the abundance of A1AR protein. A1AR controls the internalization of pathogenic tau seeds via ASAP1-regulated macropinocytosis.
